# An oncolytic virus delivering tumor-irrelevant bystander T cell epitopes induces anti-tumor immunity and potentiates cancer immunotherapy

**DOI:** 10.1038/s43018-024-00760-x

**Published:** 2024-04-12

**Authors:** Xiangyu Chen, Jing Zhao, Shuai Yue, Ziyu Li, Xiang Duan, Yao Lin, Yang Yang, Junjian He, Leiqiong Gao, Zhiwei Pan, Xiaofan Yang, Xingxing Su, Min Huang, Xiao Li, Ye Zhao, Xuehui Zhang, Zhirong Li, Li Hu, Jianfang Tang, Yaxing Hao, Qin Tian, Yifei Wang, Lifan Xu, Qizhao Huang, Yingjiao Cao, Yaokai Chen, Bo Zhu, Yan Li, Fan Bai, Guozhong Zhang, Lilin Ye

**Affiliations:** 1 Institute of Immunological Innovation and Translation, Chongqing Medical University, Chongqing, China; 2 Changping Laboratory, Beijing, China; 3 Key Laboratory of Animal Epidemiology of the Ministry of Agriculture, College of Veterinary Medicine, China Agricultural University, Beijing, China; 4 Institute of Immunology, Third Military Medical University, Chongqing, China; 5 Cancer Center, Daping Hospital and Army Medical Center of PLA, Third Military Medical University, Chongqing, China; 6 Biomedical Pioneering Innovation Center (BIOPIC), School of Life Sciences, Peking University, Beijing, China; 7 Beijing Advanced Innovation Center for Genomics, Peking University, Beijing, China; 8 The State Key Laboratory of Pharmaceutical Biotechnology, National Resource Center for Mutant Mice, MOE Key Laboratory of Model Animals for Disease Study, MOE Engineering Research Center of Protein and Peptide Medicine, Chemistry and Biomedicine Innovation Center, Model Animal Research Center, Medical School of Nanjing University, Nanjing, China; 9 Guangdong Provincial Key Laboratory of Immune Regulation and Immunotherapy, School of Laboratory Medicine and Biotechnology, Southern Medical University, Guangzhou, China; 10 Dermatology Hospital, Southern Medical University, Guangzhou, China; 11 Department of Hepatobiliary Surgery, Southwest Hospital, Third Military Medical University, Chongqing, China; 12 Department of Infectious Diseases, Chongqing Public Health Medical Center, Chongqing, China; 13 Institute of Cancer, Xinqiao Hospital, Third Military Medical University, Chongqing, China

**Keywords:** Cancer, Cancer immunotherapy, Tumour immunology

## Abstract

Tumor-specific T cells are crucial in anti-tumor immunity and act as targets for cancer immunotherapies. However, these cells are numerically scarce and functionally exhausted in the tumor microenvironment (TME), leading to inefficacious immunotherapies in most patients with cancer. By contrast, emerging evidence suggested that tumor-irrelevant bystander T (T_BYS_) cells are abundant and preserve functional memory properties in the TME. To leverage T_BYS_ cells in the TME to eliminate tumor cells, we engineered oncolytic virus (OV) encoding T_BYS_ epitopes (OV-BYTE) to redirect the antigen specificity of tumor cells to pre-existing T_BYS_ cells, leading to effective tumor inhibition in multiple preclinical models. Mechanistically, OV-BYTE induced epitope spreading of tumor antigens to elicit more diverse tumor-specific T cell responses. Remarkably, the OV-BYTE strategy targeting human severe acute respiratory syndrome coronavirus 2 (SARS-CoV-2)-specific T cell memory efficiently inhibited tumor progression in a human tumor cell-derived xenograft model, providing important insights into the improvement of cancer immunotherapies in a large population with a history of SARS-CoV-2 infection or coronavirus disease 2019 (COVID-19) vaccination.

## Abstract (web summary)

Ye and colleagues show that an oncolytic virus that delivers tumor-irrelevant bystander T cell epitopes to tumor cells can exploit the abundant population of bystander T cells in the tumor for anti-tumor immunity and potentiate cancer immunotherapy.

## Main

After acute infections or vaccinations, memory T (T_MEM_) cells form and provide long-term protection against cognate pathogens^1^. By contrast, the functional exhaustion of T cells has been well characterized in many chronic infections and cancers^2^. Tumor-infiltrating CD8^+^ T cells that specifically recognize tumor antigens manifest typical exhaustion-associated phenotypes, including progressive loss of effector functions, proliferative capacities and memory potential, while having elevated and sustained expression of a cluster of inhibitory immune checkpoint receptors (for example, PD-1, CTLA-4 and TIM-3)^2^. Blocking antibodies targeting these inhibitory immune checkpoint receptors, termed immune checkpoint blockade (ICB), can reinvigorate exhausted CD8^+^ T (T_EX_) cells in the TME, which thus lays the foundation for the clinical effects of ICB. Although ICB therapies targeting PD-1–programmed cell death ligand 1 (PD-L1) interaction have documented durable remissions in a subset of patients with cancer, the overall response rates remain modest and even inert in certain cancer types^3^, which might be partially explained by the fact that ICB therapies do not fundamentally alter the exhaustion-inherited epigenetic program in T_EX_ cells and fail to reprogram these cells to differentiate into T_MEM_ cells^4,5^.

Accumulating evidence has suggested that the specific recognition of tumor-derived antigens is restricted to a minor proportion of tumor-infiltrating T cells, while the vast majority of tumor-infiltrating T cells recognize tumor-irrelevant antigens, especially common viral antigens, and are thus defined as ‘T_BYS_ cells’ (refs. ^6–15^). Tumor-infiltrating T_BYS_ cells were reported to exhibit T_MEM_-associated phenotypes^9,12,14,15^; however, their detailed differentiation states are not well characterized. Particularly, it remains unknown whether CD8^+^ T_BYS_ cells and CD4^+^ ones share similar memory traits in the TME. Furthermore, owing to the overwhelming number and function of T_BYS_ cells compared to tumor-specific T_EX_ cells in the TME, it would be of great therapeutic value if T_BYS_ cells in situ could be repurposed to eliminate tumor cells.

OV represents a highly versatile platform in delivering various payloads into tumor cells^16,17^. Many DNA or RNA viruses with potent tumor tropism have been proposed as vectors for OV immunotherapies, such as herpes simplex virus (HSV), adenovirus and Newcastle disease virus (NDV)^16,17^. To this day, OV immunotherapies have been investigated in ~100 reported clinical trials, in which the modified HSV encoding granulocyte–macrophage colony-stimulating factor (termed T-VEC) has been approved by the US Food and Drug Administration^17^ and another triple-mutated HSV (termed as G47Δ) has been approved to treat recurrent glioblastoma in Japan^18^. It remains unknown whether OV can be exploited to drive tumor cells to present T_BYS_ cell-specific antigens, thus causing tumor cells to be eradicated by T_BYS_ cells of functional memory properties in the TME.

## Results

### Features of tumor-infiltrating virus-specific T_BYS_ cells

**Fig. 1:**
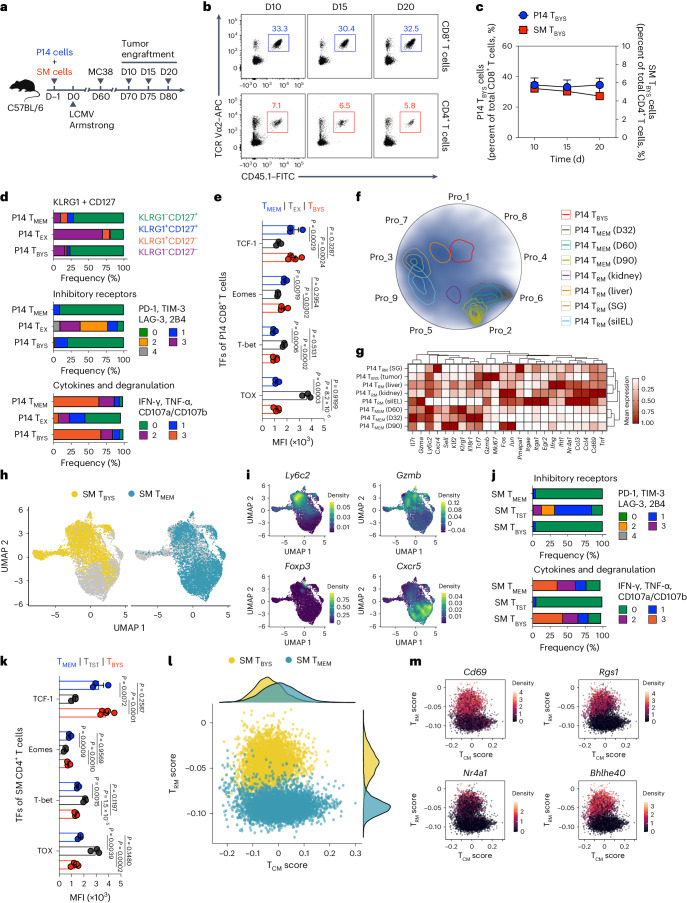
Features of tumor-infiltrating virus-specific T_BYS_ cells. **a**, Schematic of the experimental design. Congenic CD45.1^+^ P14 CD8^+^ T cells and CD45.1^+^ SM CD4^+^ T cells were adoptively transferred into naive C57BL/6 recipients (CD45.2^+^), which were then infected with LCMV Armstrong and engrafted with MC38 cells on day (D)60 after infection. On days 10, 15 and 20 after tumor engraftment, tumor-infiltrating P14 and SM cells were analyzed. **b**, Flow cytometry analysis of MC38 tumor-infiltrating CD8^+^ (top) and CD4^+^ (bottom) T cells on days 10, 15 and 20 after tumor engraftment. Numbers adjacent to the outlined areas indicate percentages of CD45.1^+^Vα2^+^ P14 cells (blue) of tumor-infiltrating CD8^+^ T cells or CD45.1^+^Vα2^+^ SM cells (red) of tumor-infiltrating CD4^+^ T cells. **c**, Frequency of P14 T_BYS_ cells of total MC38 tumor-infiltrating CD8^+^ T cells (indicated by blue dots, *n* = 5 mice (day 10) or *n* = 4 mice (day 15 and day 20)) and frequency of SM T_BYS_ cells of total MC38 tumor-infiltrating CD4^+^ T cells (indicated by red cubes, *n* = 5 mice (day 10) or *n* = 4 mice (day 15 and day 20)) at the indicated time points. **d**, Frequencies of P14 T_MEM_, P14 T_EX_ and P14 T_BYS_ cells that express KLRG1 and/or CD127 (top; *n* = 4 mice (P14 T_MEM_), *n* = 3 mice (P14 T_EX_) or *n* = 5 mice (P14 T_BYS_)), coexpress the indicated number of inhibitory receptors (PD-1, TIM-3, LAG-3 and 2B4) (middle; *n* = 5 mice (P14 T_MEM_), *n* = 3 mice (P14 T_EX_) or *n* = 4 mice (P14 T_BYS_)) or coexpress the indicated number of cytokines (IFN-γ and tumor necrosis factor (TNF)-α) and cytotoxic degranulation markers (CD107a and CD107b) (bottom; *n* = 5 mice (P14 T_MEM_), *n* = 3 mice (P14 T_EX_) or *n* = 3 mice (P14 T_BYS_)). **e**, Comparison of TCF-1, Eomes, T-bet and TOX expression levels among P14 T_MEM_ (*n* = 3 mice), P14 T_EX_ (*n* = 3 mice) and P14 T_BYS_ cells (*n* = 5 mice). MFI, mean fluorescence intensity. **f**, A Radviz projection of P14 T_MEM_, P14 T_RM_ (kidney, liver, salivary gland (SG) and small intestine intra-epithelial lymphocyte (siIEL)) and P14 T_BYS_ cells based on nine gene expression programs (Pro_1–Pro_9) inferred by consensus non-negative matrix factorization. A total of 38,880 P14 cells passed quality control. **g**, Heatmap showing selected DEGs in various P14 cells. **h**, UMAPs of SM T_BYS_ (left) and SM T_MEM_ cells (right). A total of 3,565 SM T_BYS_ cells are colored in yellow, and a total of 5,648 SM T_MEM_ cells are colored in blue. **i**, UMAPs showing expression levels of *Ly6c2*, *Gzmb*, *Foxp3* and *Cxcr5* in SM T_BYS_ and SM T_MEM_ cells. **j**, Frequency of SM T_MEM_, SM T_TST_ and SM T_BYS_ cells that coexpress the indicated number of inhibitory receptors (PD-1, TIM-3, LAG-3 and 2B4) (top; *n* = 3 mice (SM T_MEM_), *n* = 3 mice (SM T_TST_) or *n* = 3 mice (SM T_BYS_)) or coexpress the indicated number of cytokines (IFN-γ and TNF-α) and cytotoxic degranulation markers (CD107a and CD107b) (bottom; *n* = 3 mice (SM T_MEM_), *n* = 3 mice (SM T_TST_) or *n* = 5 mice (SM T_BYS_)). **k**, Comparison of TCF-1, Eomes, T-bet and TOX expression levels among SM T_MEM_ (*n* = 3 mice), SM T_TST_ (*n* = 3 mice) and SM T_BYS_ (*n* = 5 mice) cells. TFs, transcription factors. **l**, Gene set scoring analysis of 3,565 SM T_BYS_ cells and 5,648 SM T_MEM_ cells. Density of cells in the T_CM_ versus T_RM_ score space annotated by cell type is depicted. **m**, Expression profiles of *Cd69*, *Rgs1*, *Nr4a1* and *Bhlhe40* of SM T_BYS_ and SM T_MEM_ cells in the T_CM_ versus T_RM_ score space. Data (**b**–**e**,**j**,**k**) are representative of two independent experiments. One-way ANOVA with Turkey’s test was used in **e**,**k**. Center values and error bars (**c**,**e**,**k**) indicate mean and s.e.m. Source data

**Extended Data Fig. 1:**
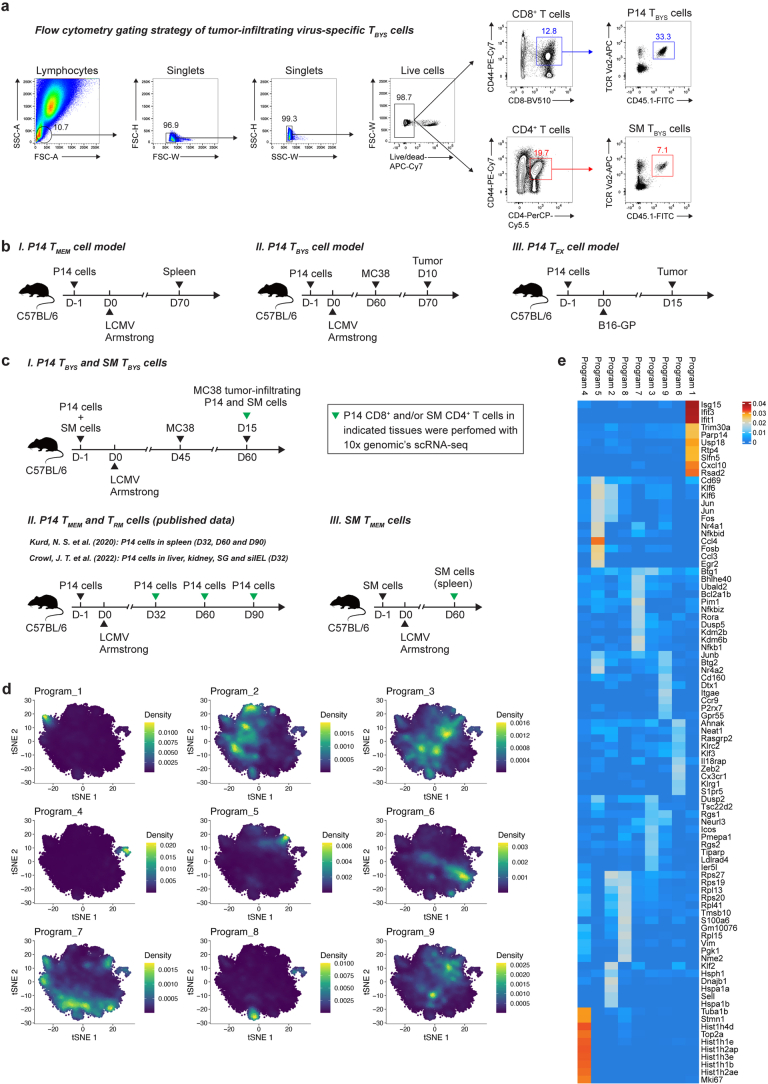
Molecular characteristics of virus-specific T_BYS_ cells. **a**, Flow cytometry gating strategy of MC38 tumor-infiltrating P14 CD8^+^ T_BYS_ cells and SM CD4^+^ T_BYS_ cells. Congenic CD45.1^+^ P14 CD8^+^ T cells and CD45.1^+^ SM CD4^+^ T cells were adoptively transferred into naïve C57BL/6 recipients (CD45.2^+^), which were then infected with LCMV Armstrong and inoculated with MC38 cells on day 60 after infection. On day 10, 15 and 20 after tumor engraftment, tumor-infiltrating P14 and SM cells were analyzed by flow cytometry. The leftmost flow cytometry data (FSC^−^A/SSC-A) showed total cells in MC38 tumors, wherein lymphocytes were gated and further gated for singlets in the adjacent two flow cytometry data (FSC-H/FSC-W and SSC-H/SSC-W). Then, live cells (negative for Live/dead-dye-APC-Cy7) were gated on singlets and further gated for CD8^+^CD44^+^ T cells or CD4^+^CD44^+^ T cells. Lastly, CD45.1^+^Vα2^+^ P14 CD8^+^ T cells and CD45.1^+^Vα2^+^ SM CD4^+^ T cells were gated on CD8^+^CD44^+^ T cells or CD4^+^CD44^+^ T cells, respectively. **b**, Schematic graph of the experimental design. (I) P14 T_MEM_ cell model: congenic CD45.1^+^ P14 CD8^+^ T cells were adoptively transferred into naïve C57BL/6 recipients (CD45.2^+^), which were then infected with LCMV Armstrong. On day 70 after infection, CD45.1^+^Vα2^+^ P14 cells (defined as CD8^+^ T_MEM_ cells) from the spleens of LCMV Armstrong-infected recipients were analyzed. (II) P14 T_BYS_ cell model: congenic CD45.1^+^ P14 CD8^+^ T cells were adoptively transferred into naïve C57BL/6 recipients (CD45.2^+^), which were then infected with LCMV Armstrong and engrafted with MC38 cells on day 60 after infection. On day 10-post tumor engraftment, CD45.1^+^Vα2^+^ P14 cells (defined as CD8^+^ T_BYS_ cells) from the MC38 tumors of LCMV Armstrong-infected recipients were analyzed. (III) P14 T_EX_ cell model: congenic CD45.1^+^ P14 CD8^+^ T cells were adoptively transferred into naïve C57BL/6 recipients (CD45.2^+^), which were then engrafted with B16-GP cells. On day 15^−^post tumor engraftment, CD45.1^+^Vα2^+^ P14 (defined as CD8^+^ T_EX_ cells) from the B16-GP tumors were analyzed. **c**, Schematic graph of the experimental design for scRNA-seq data. (I) P14 T_BYS_ and SM T_BYS_ cell model: congenic CD45.1^+^ P14 CD8^+^ T cells and CD45.1^+^ SM CD4^+^ T cells were adoptively transferred into naïve C57BL/6 recipients (CD45.2^+^), which were then infected with LCMV Armstrong and engrafted with MC38 cells on day 45 after infection. On day 15-post tumor engraftment, CD45.1^+^Vα2^+^ P14 cells and CD45.1^+^Vα2^+^ SM cells were isolated from the MC38 tumors and performed with scRNA-seq library preparation. (II) P14 T_MEM_ and T_RM_ cell model: congenic CD45.1^+^ P14 CD8^+^ T cells were adoptively transferred into naïve C57BL/6 recipients (CD45.2^+^), which were then infected with LCMV Armstrong. On day 32-post infection, CD45.1^+^Vα2^+^ P14 cells were isolated from the spleens, livers, kidneys, SGs and siIELs of recipients and performed with scRNA-seq library preparation. On days 60 and 90 after infection, CD45.1^+^Vα2^+^ P14 cells were isolated from the spleens of recipients and performed with scRNA-seq library preparation. These P14 cell datasets were exacted from the external scRNA-seq datasets (GSE182276 and GSE131847). (III) SM T_MEM_ cell model: congenic CD45.1^+^ SM CD4^+^ T cells were adoptively transferred into naïve C57BL/6 recipients (CD45.2^+^), which were then infected with LCMV Armstrong. On day 60 after infection, CD45.1^+^Vα2^+^ SM cells were isolated from the spleens of recipients and performed with scRNA-seq library preparation. **d**, tSNEs showing activities of 9 gene expression programs in P14 T_MEM_, P14 T_RM_ and P14 T_BYS_ cells inferred by consensus non-negative matrix factorization (cNMF), with 38,880 cells in total passing quality control. **e**, Heatmap showing the top 10 differential expressed genes in each program in d. Source data

Although virus-specific T_BYS_ cells have been reported by accumulating evidence^6–15^, a detailed portrait of these specialized T cells, especially CD4^+^ T_BYS_ cells, is lacking. We thus sought to analyze the kinetics, molecular characteristics and lineage relationship with other memory types of both CD8^+^ and CD4^+^ T_BYS_ populations in the TME. To this end, congenic naive (CD45.1^+^CD44^lo^CD62L^hi^) P14 CD8^+^ T cells recognizing the lymphocytic choriomeningitis virus (LCMV) glycoprotein (GP) epitope H-2D^b^GP_33–41_ and congenic naive (CD45.1^+^CD44^lo^CD62L^hi^) SMARTA (SM) CD4^+^ T cells recognizing the LCMV GP epitope I-A^b^GP_66–77_ were adoptively transferred into C57BL/6 recipients (CD45.2^+^), which were then infected with LCMV Armstrong to establish acute viral infection and resultant virus-specific P14 T_MEM_ and SM T_MEM_ cells. On day 60 after infection, recipients were subcutaneously engrafted with syngeneic MC38 colon adenocarcinoma cells and tumor-infiltrating P14 CD8^+^ and SM CD4^+^ T cells, and T_BYS_ populations were analyzed on days 10, 15 and 20 after tumor engraftment (Fig. 1a and Extended Data Fig. 1a). As shown, frequencies of tumor-infiltrating P14 T_BYS_ and SM T_BYS_ cells remained stable at the different indicated time points (Fig. 1b,c), suggesting that the infiltrating virus-specific T_BYS_ cells were not altered at the population level during tumor progression.

Next, we set out to compare the molecular characteristics of virus-specific CD8^+^ T_BYS_ cells with their counterparts recognizing the same epitope but induced to differentiate into T_MEM_ or T_EX_ cells. Accordingly, congenic naive P14 CD8^+^ T cells were adoptively transferred into C57BL/6 recipients, which were infected with the LCMV Armstrong strain to induce the differentiation of P14 T_MEM_ cells. Furthermore, a fraction of these infected recipients was subcutaneously engrafted with MC38 cells on day 60 after infection, and tumor-infiltrating P14 T_BYS_ cells were analyzed on day 10 after tumor engraftment. In the scenario of tumor-specific T_EX_ cells, congenic naive P14 CD8^+^ T cells were adoptively transferred into C57BL/6 recipients, which were then subcutaneously engrafted with syngeneic B16F10 melanoma cells expressing the LCMV GP (hereafter referred to as B16-GP cells)^19^ and killed on day 15 after tumor engraftment to analyze tumor-specific P14 T_EX_ cells (Extended Data Fig. 1b).

Resembling P14 T_MEM_ cells, we found that P14 T_BYS_ cells are mainly populated by the killer cell lectin-like receptor G1 (KLRG1)^lo^CD127^hi^ subset^1^ (Fig. 1d). Furthermore, P14 T_BYS_ cells retain an expression pattern of transcription factors similar to that of P14 T_MEM_ cells^1^, as exemplified by high expression of TCF-1 and Eomes and low expression of T-bet (Fig. 1e). In contrast to T_MEM_ cells, T_EX_ cells are characterized by coexpression of multiple inhibitory receptors, impaired cytokine secretion and the transcription factor TOX associated with T cell exhaustion^2,20–23^. Although preserved in the immunosuppressive TME, P14 T_BYS_ cells show limited expression of inhibitory receptors and TOX and exhibit polyfunctional cytokine secretion upon ex vivo re-stimulation (Fig. 1d,e). Thus, these findings confirm that CD8^+^ T_BYS_ cells phenotypically and functionally resemble CD8^+^ T_MEM_ cells but not CD8^+^ T_EX_ cells.

**Extended Data Fig. 2:**
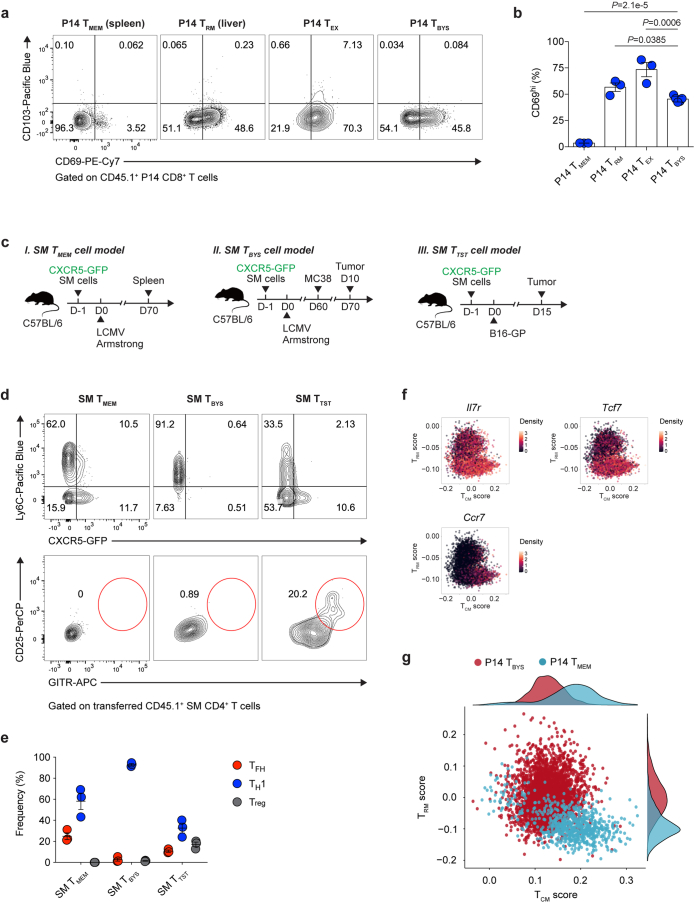
Differentiation patterns of tumor-infiltrating virus-specific T_BYS_ cells. **a**, Flow cytometry analysis of CD69 and CD103 expressions in splenic P14 T_MEM_ cells, P14 T_RM_ cells (liver), tumor-specific P14 T_EX_ cells and tumor-infiltrating P14 T_BYS_ cells. **b**, Frequencies of CD69^hi^ cells in P14 T_MEM_ cells (n = 3 mice), P14 T_RM_ cells (n = 3 mice), P14 T_EX_ cells (n = 3 mice) and P14 T_BYS_ cells (n = 5 mice) in a. **c**, Schematic graph of the experimental design. (I) SM T_MEM_ cell model: CD45.1^+^ CXCR5-GFP SM CD4^+^ T cells were adoptively transferred into naïve C57BL/6 recipients (CD45.2^+^), which were then infected with LCMV Armstrong. On day 70 after infection, splenic SM T_MEM_ cells were analyzed. (II) SM T_BYS_ cell model: congenic CD45.1^+^ CXCR5-GFP SM CD4^+^ T cells were adoptively transferred into naïve C57BL/6 recipients (CD45.2^+^), which were then infected with LCMV Armstrong and engrafted with MC38 cells on day 60 after infection. On day 10^−^post tumor engraftment, tumor-infiltrating SM T_BYS_ cells were analyzed. (III) SM T_TST_ cell model: congenic CXCR5-GFP SM CD4^+^ T cells were adoptively transferred into naïve C57BL/6 recipients (CD45.2^+^), which were then engrafted with B16-GP cells. On day 15-post tumor engraftment, SM T_TST_ cells were analyzed. **d**, Flow cytometry analysis of CXCR5/Ly6C expressions (top panel) and GITR/CD25 expressions (bottom panel) in SM T_MEM_, SM T_BYS_ and SM T_TST_ cells. **e**, Frequencies of CXCR5^+^Ly6C^-^ T_FH_, CXCR5^-^Ly6C^+^ T_H_1 and GITR^+^CD25^+^ T_reg_ cells in SM T_MEM_ (n = 3 mice), SM T_BYS_ (n = 3 mice) and SM T_TST_ (n = 3 mice) cells. **f**, Expression profiles of *Il7r*, *Tcf7* and *Ccr7* of 3,565 SM T_BYS_ cells and 5,648 SM T_MEM_ cells in T_CM_ versus T_RM_ score space. **g**, Geneset scoring analysis of 3,322 P14 T_BYS_ cells and 764 P14 T_MEM_ cells. Density of cells in T_CM_ versus T_RM_ score space annotated by cell type are depicted. All the data are representative of two independent experiments. One-way ANOVA with Turkey’s test was used in b. Error bars (b, e) indicate s.e.m. Source data

To further define the molecular traits of CD8^+^ T_BYS_ cells, we performed single-cell RNA sequencing (scRNA-seq) of P14 T_BYS_ cells from the TME of MC38-engrafted mice. Conventional P14 T_MEM_ and tissue-resident memory (T_RM_) cells from external scRNA-seq datasets^24,25^ were introduced as controls (Extended Data Fig. 1c). A Radviz projection based on nine gene expression programs inferred by consensus non-negative matrix factorization clearly distinguished P14 T_MEM_ cells from P14 T_RM_ cells of various non-lymphoid tissues (Fig. 1f and Extended Data Fig. 1d,e). Importantly, P14 T_BYS_ cells were drawn toward the groups of P14 T_RM_ cells (Fig. 1f), likely representing the acquisition of certain T_RM_ cell features. Further unbiased hierarchical clustering confirmed that P14 T_BYS_ cells are in close proximity with P14 T_RM_ cells and highlighted by cytolytic activity (*Gzmb*, *Tnf*) and tissue residency (*Cd69*, *Nr4a1*)^26^ (Fig. 1g). The potential residency of P14 T_BYS_ cells was also evidenced by high expression of CD69 protein (Extended Data Fig. 2a,b). Therefore, these findings suggest T_RM_ cell features of CD8^+^ T_BYS_ cells.

Next, we aimed to trace the differentiation of virus-specific CD4^+^ T_BYS_ cells by performing scRNA-seq of SM T_BYS_ cells from the MC38 TME with conventional SM T_MEM_ cells as controls (Extended Data Fig. 1c). Consistent with previous studies^27,28^, SM T_MEM_ cells adopt a bifurcation of follicular helper T cells (*Cxcr5*) and type 1 helper T (T_H_1) cells (*Ly6c2*, *Gzmb*) (Fig. 1h,i). Instead of adopting a follicular helper T/T_H_1 bifurcation, we found that SM T_BYS_ cells were predominantly fated toward the T_H_1 lineage (Fig. 1h,i). Indeed, T_H_1-type SM T_BYS_ cell differentiation was further evidenced by abundant Ly6C protein but rare expression of C–X–C motif chemokine receptor (CXCR)5 (Extended Data Fig. 2c–e). Furthermore, similar to P14 T_BYS_ cells, SM T_BYS_ cells also showed high expression of TCF-1 and Eomes and low expression of T-bet (Fig. 1k), suggesting the retention of memory properties. In addition, SM T_BYS_ cells showed no features related to exhaustion, including multiple inhibitory receptors^29^ and TOX^30^, as compared to tumor antigen-specific T (T_TST_) cells (Fig. 1j,k); instead, SM T_BYS_ cells exhibit a substantial capacity to secrete multiple cytokines upon ex vivo re-stimulation (Fig. 1j). These results define the memory properties of T_H_1-type CD4^+^ T_BYS_ cells in the TME.

Previous studies stated distinct molecular signatures between central memory T (T_CM_) and T_RM_ cells^31,32^. We therefore examined the enrichment for T_CM_ and T_RM_ cell gene signatures in SM T_BYS_ cells, with conventional SM T_MEM_ cells being introduced as controls, to explore the effector–memory state in the SM T_BYS_ population. We found a largely overlaid T_CM_ signature between SM T_BYS_ and SM T_MEM_ cells, with a noticeable dissection in the T_RM_ signature (Fig. 1l). Indeed, genes related to T_RM_ cells (for example, *Cd69*, *Rgs1*, *Nr4a1* and *Bhlhe40*) were highly expressed in SM T_BYS_ cells relative to those in SM T_MEM_ cells (Fig. 1m); meanwhile, genes related to T_CM_ cells (for example, *Il7r*, *Tcf7* and *Ccr7*) were largely overlapping between SM T_BYS_ and SM T_MEM_ cells (Extended Data Fig. 2f). Distinct from SM T_BYS_ cells, P14 T_BYS_ cells were dominated by the gene signature of T_RM_ cells rather than that of T_CM_ cells (Extended Data Fig. 2g). Thus, these findings highlight a unique hybrid gene signature consisting of both T_CM_ and T_RM_ cell features in CD4^+^ T_BYS_ cells but not in CD8^+^ T_BYS_ cells.

**Extended Data Fig. 3:**
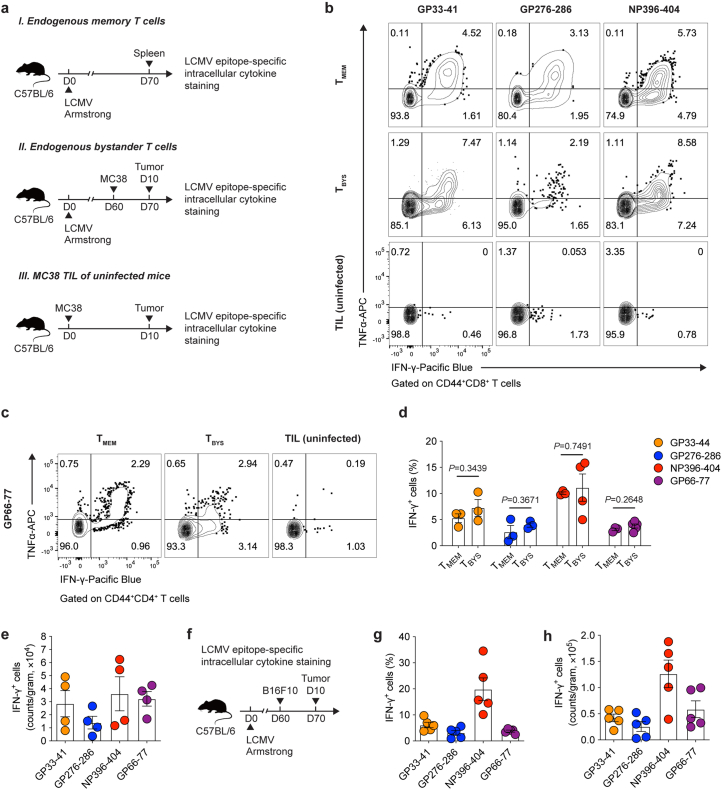
Characteristics of tumor-infiltrating endogenous virus-specific T_BYS_ cells. **a**, Schematic graph of the experimental design. (I) Endogenous T_MEM_ cells: naïve C57BL/6 mice were infected with LCMV Armstrong and splenic LCMV-specific T cells were determined by peptide stimulation assay on day 70 after infection. (II) Endogenous T_BYS_ cells: naïve C57BL/6 mice were infected with LCMV Armstrong and engrafted with MC38 cells on day 60 after infection. On day 10 after tumor engraftment, tumor-infiltrating LCMV-specific T cells were determined by peptide stimulation assay. (III) MC38 TIL of uninfected mice: naïve C57BL/6 mice were engrafted with MC38 cells and tumor-infiltrating T cells were determined by peptide stimulation assay on day 10 after tumor engraftment. **b**, Flow cytometry analysis of IFN-γ and TNF-α expressions in CD8^+^ T cells originating from spleens of LCMV Armstrong-infected mice, MC38 tumors of LCMV Armstrong-infected mice or MC38 tumors of uninfected mice under the condition of LCMV GP_33–41_ or GP_276–286_ or NP_396–404_ peptide stimulation. **c**, Flow cytometry analysis of IFN-γ and TNF-α expressions in CD4^+^ T cells originating from spleens of LCMV Armstrong-infected mice, MC38 tumors of LCMV Armstrong-infected mice or MC38 tumors of uninfected mice under the condition of LCMV GP_66–77_ peptide stimulation. **d**, Frequencies of IFN-γ^+^ cells in CD8^+^ T cells (LCMV GP_33–41_ or GP_276–286_ or NP_396–404_ stimulation) or CD4^+^ T cells (LCMV GP_66–77_ stimulation) originating from spleens of LCMV Armstrong-infected mice (memory) or MC38 tumors of LCMV Armstrong-infected mice (bystander). Memory (n = 3 (GP_33–41_), 3 (GP_276–286_), 3 (NP_396–404_), or 3 (GP_66–77_) mice), bystander (n = 4 (GP_33–41_), 3 (GP_276–286_), 4 (NP_396–404_), or 5 (GP_66–77_) mice). **e**, Numbers of IFN-γ-expressing CD8^+^ T cells (LCMV GP_33–41_ or GP_276–286_ or NP_396–404_ stimulation) or IFN-γ-expressing CD4^+^ T cells (LCMV GP_66–77_ stimulation) originating from MC38 tumors of LCMV Armstrong-infected mice. n = 4 (GP_33–41_), 4 (GP_276–286_), 4 (NP_396–404_), or 4 (GP_66–77_) mice. **f**, Schematic graph of the experimental design. Naïve C57BL/6 mice were infected with LCMV Armstrong and engrafted with B16F10 cells on day 60 after infection. On day 10 after tumor engraftment, tumor-infiltrating LCMV-specific T cells were determined by peptide stimulation assay. **g**, **h**, Frequencies (g) and numbers (h) of IFN-γ-expressing CD8^+^ T cells (LCMV GP_33–41_ or GP_276–286_ or NP_396–404_ stimulation) or IFN-γ-expressing CD4^+^ T cells (LCMV GP_66–77_ stimulation) originating from B16F10 tumors of LCMV Armstrong-infected mice. n = 5 (GP_33–41_), 5 (GP_276–286_), 5 (NP_396–404_), or 5 (GP_66–77_) mice in g and h. All the data are representative of two independent experiments. Two-tailed unpaired Student’s *t*-test was used in d. Error bars (d, e, g, h) indicate s.e.m. Source data

We further explored endogenous virus-specific T_BYS_ cells from MC38 tumors engrafted in LCMV-infected mice by quantifying interferon (IFN)-γ-expressing cells upon ex vivo stimulation with cognate viral peptides (Extended Data Fig. 3a). Consistently, we observed the presence of endogenous CD8^+^ T_BYS_ cells specific to LCMV GP_33–41_, LCMV GP_276–286_ and LCMV nucleoprotein (NP)_396–404_ and endogenous CD4^+^ T_BYS_ cells specific to LCMV GP_66–77_ in the MC38 tumors engrafted in LCMV-infected mice (Extended Data Fig. 3b–e). In addition to the MC38 tumor model, endogenous LCMV-specific T_BYS_ cells were also found in the TME of B16F10-engrafted mice (Extended Data Fig. 3f–h). In sum, these findings highlight the functional memory characteristics of virus-specific CD8^+^ and CD4^+^ T_BYS_ cells in the TME.

### OV-BYTE therapy elicits anti-tumor response

**Extended Data Fig. 4:**
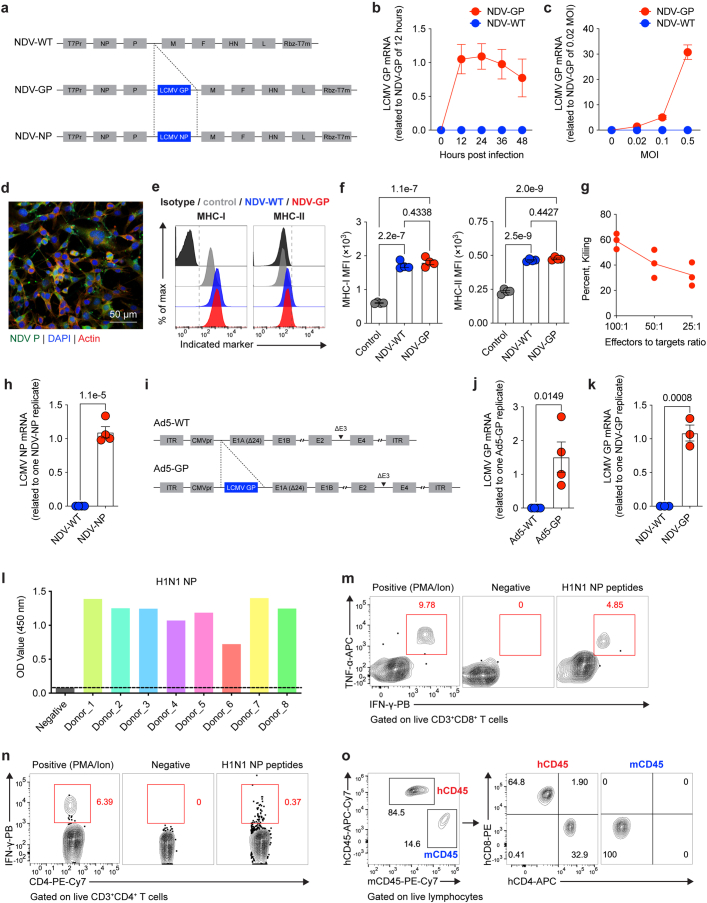
Construction of oncolytic viruses expressing LCMV viral proteins and humanized mice. **a**, Schematic graph of NDV rSG10 (NDV-WT) or modified NDV rSG10 expressing LCMV GP (NDV-GP) or LCMV NP (NDV-NP). **b**, Relative quantification of LCMV GP mRNA in MC38 cells at indicated timepoints (12, 24, 36 and 48 hours) after initial exposure to the 0.1 multiplicity of infection (MOI) of NDV-WT (n = 3 wells of cultured cells with biological independence) or NDV-GP (n = 3 wells of cultured cells with biological independence). **c**, Relative quantification of LCMV GP mRNA in MC38 cells at 24 hours after initial exposure to the indicated MOIs (0, 0.02, 0.1 and 0.5) of NDV-WT (n = 3 wells of cultured cells with biological independence) or NDV-GP (n = 3 wells of cultured cells with biological independence). **d**, Immunofluorescence microscopy of MC38 cells pre-treated with NDV-GP at MOI of 0.5 for 24 hours. Scale bar, 50 μm. Red indicates actin, blue indicates DAPI and green indicates NDV P protein. **e**, Flow cytometry analysis of MHC-I (left) and MHC-II (right) expressions in MC38 cells infected for 24 hours with NDV-WT or NDV-GP at MOI of 0.5 or in uninfected MC38 cells. **f**, MHC-I (left) and MHC-II (right) expression levels among MC38 cells in e. Control (n = 4 wells of cultured cells with biological independence), NDV-WT (n = 4 wells of cultured cells with biological independence) and NDV-GP (n = 4 wells of cultured cells with biological independence). **g**, *Ex vitro* killing efficiency of LCMV-specific CD8^+^ T cells to NDV-GP-infected MC38 cells. The n value was 3 (wells of cultured cells with biological independence) in each group. **h**, Relative quantification of LCMV NP mRNA in MC38 cells after 24-hour exposure to 0.5 MOI of NDV-WT (n = 4 wells of cultured cells with biological independence) or NDV-NP (n = 4 wells of cultured cells with biological independence). **i**, Schematic graph of Ad5 (Ad5-WT) or modified Ad5 expressing LCMV GP (Ad5-GP). **j**, Relative quantification of LCMV GP mRNA in MC38 cells after 24-hour exposure to 0.5 MOI of Ad5-WT (n = 4 wells of cultured cells with biological independence) or Ad5-GP (n = 4 wells of cultured cells with biological independence). **k**, Relative quantification of LCMV GP mRNA in B16F10 cells after 24-hour exposure to 0.5 MOI of NDV-WT (n = 3 wells of cultured cells with biological independence) or NDV-GP (n = 3 wells of cultured cells with biological independence). **l**, ELISA binding assays of healthy donor sera to ELISA plate coating of H1N1 NP protein. **m**, Flow cytometry analysis of CD3^+^CD8^+^ T cells from the PBMCs of donor_1 under the condition of PMA/Ion stimulation (positive control) or DMSO (negative control) or H1N1 NP peptides. The numbers adjacent to the outlined areas indicate the frequencies of IFN-γ^+^TNF-α^+^ cells in CD3^+^CD8^+^ T cells. **n**, Flow cytometry analysis of CD3^+^CD4^+^ T cells from the PBMCs of donor_1 under the condition of PMA/Ion stimulation (positive control) or DMSO (negative control) or H1N1 NP peptides. The numbers adjacent to the outlined areas indicate the frequencies of IFN-γ^+^ cells in CD3^+^CD4^+^ T cells. **o**, Flow cytometry analysis of PBMCs of humanized NCG mice. Left panel, the numbers adjacent to the outlined areas indicate the proportions of human CD45^+^ (hCD45) cells or mouse CD45^+^ (mCD45) cells in live lymphocytes; right panel, the numbers adjacent to the outlined areas indicate the proportions of human CD8^+^ (hCD8) or human CD4^+^ (hCD4) T cells in hCD45 or mCD45 cells, respectively. Data in b-h, j-o are representative of at least two independent experiments. One-way ANOVA with Turkey’s test was used in f. Two-tailed unpaired Student’s *t*-test was used in h, j, k. Error bars (b, c, f, h, j, k) indicate s.e.m. Source data

Given the abundant memory pool of virus-specific T_BYS_ cells in the TME, we hypothesized that OV-mediated delivery of T_BYS_ cell epitopes (hereafter referred to as OV-BYTE) to tumor cells might redirect the cytotoxicity of T_BYS_ cells toward tumor cells and thus curtail tumor progression. For this purpose, the gene encoding LCMV GP was inserted into an NDV rSG10 strain (referred to as NDV wild type (WT)) and thus termed NDV-GP (Extended Data Fig. 4a). To assess the ability of NDV-GP to infect and deliver LCMV GP expression in tumor cells, we infected MC38 cells with NDV-GP and then detected LCMV GP expression in the infected MC38 cells. In accordance with the previously reported highly efficient transgene delivery of oncolytic NDV^33,34^, we found that NDV-GP substantially infected MC38 cells and expressed the LCMV GP transgene (Extended Data Fig. 4b–d). Furthermore, we also found enhanced expression of major histocompatibility complex (MHC)-I and MHC-II molecules in NDV-GP-infected tumor cells and specific killing of these tumor cells by LCMV GP-specific T cells (Extended Data Fig. 4e–g), indicating MHC presentation of LCMV GP epitopes by NDV-GP-infected tumor cells to LCMV GP-specific T cells.

**Fig. 2:**
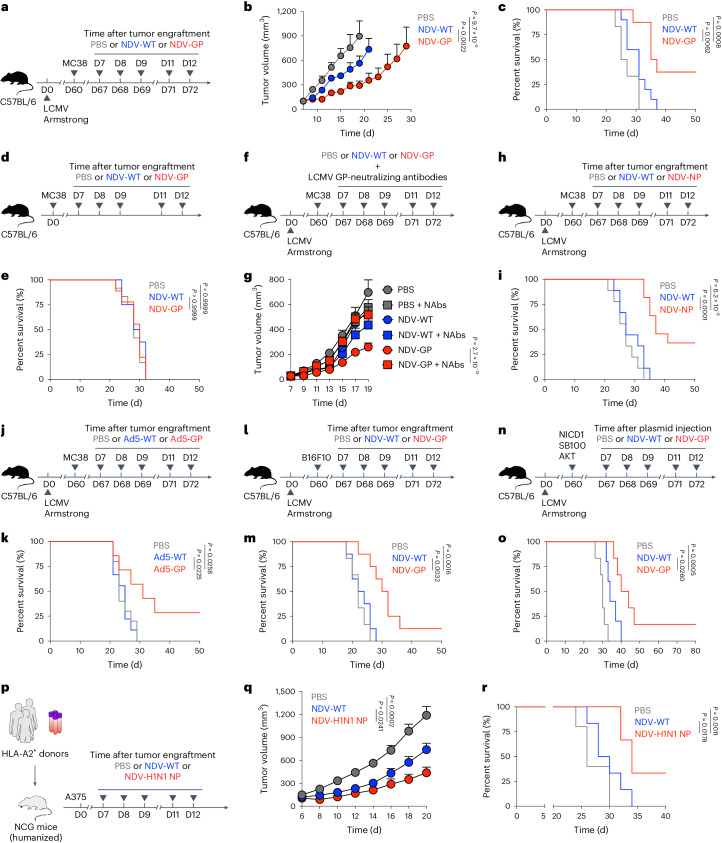
OV-mediated delivery of T_BYS_ cell epitopes to solid tumor cells elicits anti-tumor response. **a**, Schematic of the experimental design for **b**,**c**. C57BL/6 mice were infected with LCMV Armstrong and engrafted with MC38 cells on day 60 after infection. On days 7, 8, 9, 11 and 12 after tumor engraftment, recipients were intratumorally administered PBS, NDV-WT or NDV-GP. **b**,**c**, Tumor growth curve (**b**) and Kaplan–Meier survival curve (**c**) of MC38 tumor-bearing mice intratumorally treated with PBS (*n* = 6 mice), NDV-WT (*n* = 10 mice) or NDV-GP (*n* = 10 mice) as described in **a**. **d**, Schematic of the experimental design for **e**. Naive C57BL/6 mice were engrafted with MC38 cells and then intratumorally administered PBS, NDV-WT or NDV-GP on days 7, 8, 9, 11 and 12 after tumor engraftment. **e**, Kaplan–Meier survival curve of MC38 tumor-bearing mice intratumorally treated with PBS (*n* = 12 mice), NDV-WT (*n* = 8 mice) or NDV-GP (*n* = 9 mice) as described in **d**. **f**, Schematic of the experimental design for **g**. LCMV Armstrong-infected C57BL/6 mice were engrafted with MC38 cells on day 60 after infection. On days 7, 8, 9, 11 and 12 after tumor engraftment, recipients were intratumorally administered PBS, NDV-WT or NDV-GP in the presence or absence of LCMV GP-neutralizing antibodies. **g**, Tumor growth curve of MC38 tumor-bearing mice intratumorally treated with PBS, NDV-WT or NDV-GP with or without LCMV GP-neutralizing antibodies (NAbs) as described in **f**. PBS (*n* = 10 (control) or *n* = 9 (neutralizing antibodies) mice), NDV-WT (*n* = 9 (control) or *n* = 9 (neutralizing antibodies) mice) and NDV-GP (*n* = 9 (control) or *n* = 12 (neutralizing antibodies) mice). **h**, Schematic of the experimental design for **i**. C57BL/6 mice were infected with LCMV Armstrong and engrafted with MC38 cells on day 60 after infection. On days 7, 8, 9, 11 and 12 after tumor engraftment, recipients were intratumorally administered PBS, NDV-WT or NDV-NP. **i**, Kaplan–Meier survival curve of MC38 tumor-bearing mice intratumorally treated with PBS (*n* = 9 mice), NDV-WT (*n* = 9 mice) or NDV-NP (*n* = 11 mice) as described in **h**. **j**, Schematic of the experimental design for **k**. C57BL/6 mice were infected with LCMV Armstrong and engrafted with MC38 cells on day 60 after infection. On days 7, 8, 9, 11 and 12 after tumor engraftment, recipients were intratumorally administered PBS, Ad5-WT or Ad5-GP. **k**, Kaplan–Meier survival curve of MC38 tumor-bearing mice intratumorally treated with PBS (*n* = 10 mice), Ad5-WT (*n* = 9 mice) or Ad5-GP (*n* = 7 mice) as described in **j**. **l**, Schematic of the experimental design for **m**. C57BL/6 mice were infected with LCMV Armstrong and engrafted with B16F10 cells on day 60 after infection. On days 7, 8, 9, 11 and 12 after tumor engraftment, recipients were intratumorally administered PBS, NDV-WT or NDV-GP. **m**, Kaplan–Meier survival curve of B16F10 tumor-bearing mice intratumorally treated with PBS (*n* = 6 mice), NDV-WT (*n* = 8 mice) or NDV-GP (*n* = 8 mice) as described in **l**. **n**, Schematic of the experimental design for **o**. C57BL/6 mice were infected with LCMV Armstrong, and three plasmids (encoding Myc-tagged NICD1, Myc-tagged AKT and hyperactive sleeping beauty transposase (SB100)) were injected into the hydrodynamic tail vein on day 60 after infection. On days 7, 8, 9, 11 and 12 after tumor engraftment, recipients were intravenously administered PBS, NDV-WT or NDV-GP. **o**, Kaplan–Meier survival curve of mice with NICD- and AKT-induced murine intrahepatic cholangiocarcinoma as described in **n**. PBS (*n* = 6 mice), NDV-WT (*n* = 5 mice) and NDV-GP (*n* = 6 mice). **p**, Schematic of the experimental design for **q**,**r**. NCG mice were engrafted with PBMCs from HLA-A2-positive donors with a history of H1N1 infection. After reconstruction of human T cells, these humanized NCG mice were engrafted with A375 cells and then intratumorally administered PBS, NDV-WT or NDV-H1N1 NP on days 7, 8, 9, 11 and 12 after tumor engraftment. **q**,**r**, Tumor growth curve (**q**) and Kaplan–Meier survival curve (**r**) of A375 tumor-bearing humanized NCG mice intratumorally treated with PBS (*n* = 5 mice), NDV-WT (*n* = 6 mice) or NDV-H1N1 NP (*n* = 5 mice) as described in **p**. All data are representative of at least two independent experiments. Two-way ANOVA was used to compare tumor growth curves in **b**,**g**,**q**. The log-rank (Mantel–Cox) test was performed to compare survival curves among groups in **c**,**e**,**i**,**k**,**m**,**o**,**r**. Center values and error bars (**b**,**g**,**q**) indicate mean and s.e.m. Source data

Next, we aimed to assess the in vivo anti-tumor activities of NDV-GP in tumor-bearing mice that have established immune memory for LCMV. To this end, naive C57BL/6 mice were infected with LCMV Armstrong, followed by subcutaneously engraftment with MC38 cells on day 60 after infection and then intratumoral treatment with phosphate-buffered saline (PBS), NDV-WT or NDV-GP (Fig. 2a). Remarkably, NDV-GP treatment delayed tumor growth more efficiently than NDV-WT treatment (Fig. 2b) and led to ~30% complete remission on day 50 after tumor engraftment (Fig. 2c). Moreover, NDV-GP treatment did not show any therapeutic advantages over NDV-WT treatment in tumor-engrafted mice without pre-existing immune memory (Fig. 2d,e); meanwhile, the therapeutic function of NDV-GP to treat MC38 tumors engrafted in LCMV-infected mice was abolished in the presence of neutralizing antibodies specific to LCMV GP proteins (Fig. 2f,g).

In parallel, we also constructed the NDV strain carrying the LCMV NP gene (referred to as NDV-NP) to treat MC38-bearing mice with LCMV immune memory (Fig. 2h and Extended Data Fig. 4a,h). Similarly, NDV-NP treatment led to significant tumor growth delays compared to NDV-WT treatment and generated ~30% complete regression (Fig. 2i). To test whether another non-NDV OV platform could also be used to target T_BYS_ cells to limit tumor progression, LCMV Armstrong-infected C57BL/6 mice were engrafted with MC38 cells and then intratumorally administered the oncolytic adenovirus serotype 5 (Ad5) that carries the LCMV GP gene (referred to here as Ad5-GP) (Fig. 2j and Extended Data Fig. 4i,j). Similarly, Ad5-GP treatment effectively slowed tumor growth and resulted in marked tumor regression (~25% complete regression) compared to control treatments (Fig. 2k).

In addition to MC38 colon adenocarcinoma, the therapeutic functions of NDV-GP were also evident in a B16F10 melanoma model of weak immunogenicity (Fig. 2l,m and Extended Data Fig. 4k). Aside from transplanted tumor models, NDV-GP administration also largely improved the survival rates of mice with LCMV memory and Notch intracellular domain (NICD)- and AKT-induced autochthonous intrahepatic cholangiocarcinoma^35^ (Fig. 2n,o), suggesting the potential efficacy of OV-BYTE therapy in different cancer types.

To more closely examine the clinical relevance of OV-BYTE therapy, we further recruited a cohort of human leukocyte antigen (HLA)-A2-positive healthy donors with a history of influenza A (H1N1) infection (Extended Data Fig. 4l). The peripheral blood mononuclear cells (PBMCs) of these donors, which contain memory CD8^+^ and CD4^+^ T cells specific to the H1N1 NP epitope (Extended Data Fig. 4m,n), were used to develop humanized mice by engraftment of human PBMCs into NCG mice (Fig. 2p). Humanized NCG mice with an appropriate reconstruction of human T cells were engrafted with HLA-A2-matched A375 human melanoma cells to make a cell line-derived xenograft (CDX). Next, these CDX mice were intratumorally administered PBS, NDV-WT or the NDV strain expressing H1N1 NP (referred to as NDV-H1N1 NP) (Fig. 2p and Extended Data Fig. 4o). Remarkably, treatment with NDV-H1N1 NP restricted tumor growth of A375 melanoma and prolonged the overall survival of CDX mice (Fig. 2q,r). Together, these data demonstrate the potential anti-tumor functionality of OV-BYTE therapy.

### OV-BYTE therapy is attributed to CD8^+^ and CD4^+^ T_BYS_ cells

**Extended Data Fig. 5:**
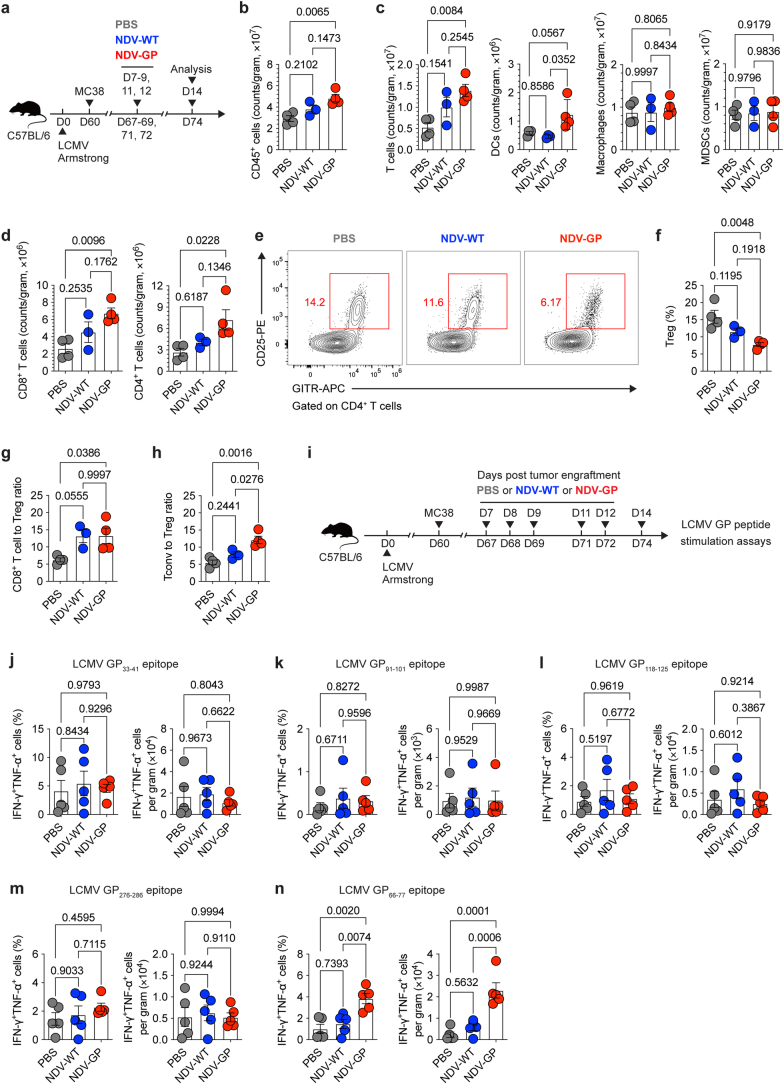
Tumor-infiltrating immune cell responses upon intratumoral administration of NDV-GP. **a**, Schematic graph of the experimental design for b-h. Naïve C57BL/6 mice were infected with LCMV Armstrong and engrafted with MC38 cells on day 60 after infection. On day 7, 8, 9, 11 and 12 after tumor engraftment, recipients were intratumorally treated with PBS or NDV-WT or NDV-GP. On day 14 after tumor engraftment, tumor-infiltrating immune cells were analyzed. **b**, Numbers of MC38 tumor-infiltrating CD45^+^ cells in mice intratumorally administrated with PBS (n = 4 mice) or NDV-WT (n = 3 mice) or NDV-GP (n = 4 mice) as described in a. **c**, Numbers of MC38 tumor-infiltrating CD45^+^CD3^+^ T cells, CD45^+^F4/80^−^CD11c^+^MHC-II^+^ dendritic cells (DCs), CD45^+^F4/80^+^CD11b^+^MHC-II^+^ macrophages, and CD45^+^F4/80^+^CD11b^+^Ly-6C^intermediate to high^ myeloid-derived suppressor cells (MDSCs) in mice intratumorally administrated with PBS (n = 4 mice) or NDV-WT (n = 3 mice) or NDV-GP (n = 4 mice). **d**, Numbers of tumor-infiltrating CD8^+^ T cells (left) and CD4^+^ T cells (right) in mice intratumorally administrated with PBS (n = 4 mice) or NDV-WT (n = 3 mice) or NDV-GP (n = 4 mice). **e**, Flow cytometry analysis of GITR and CD25 expressions in tumor-infiltrating CD4^+^ T cells in mice intratumorally administrated with PBS or NDV-WT or NDV-GP. The numbers adjacent to the outlined areas indicate the percentages of GITR^+^CD25^+^ T_reg_ cells in CD4^+^ T cells. **f**, Frequencies of tumor-infiltrating GITR^+^CD25^+^ T_reg_ cells in CD4^+^ T cells. PBS (n = 4 mice), NDV-WT (n = 3 mice), and NDV-GP (n = 4 mice). **g**, Ratios of tumor-infiltrating CD8^+^ T cells to T_reg_ cells. PBS (n = 4 mice), NDV-WT (n = 3 mice), and NDV-GP (n = 4 mice). **h**, Ratios of tumor-infiltrating T_conv_ cells to T_reg_ cells. PBS (n = 4 mice), NDV-WT (n = 3 mice), and NDV-GP (n = 4 mice). **i**, Schematic graph of the experimental design for j-n. Naïve C57BL/6 mice were infected with LCMV Armstrong and engrafted with MC38 cells on day 60 after infection. On day 7, 8, 9, 11 and 12 after tumor engraftment, recipients were intratumorally administrated with PBS or NDV-WT or NDV-GP. On day 14 after tumor engraftment, LCMV-specific T cells were determined by peptide stimulation assays in MC38 tumors of mice in each group. **j-m**, Frequencies and numbers of IFN-γ^+^TNF-α^+^ cells in tumor-infiltrating CD8^+^ T cells under the condition of LCMV GP_33–41_ (j), GP_91–101_ (k), GP_118–125_ (l) and GP_276–286_ (m) stimulation. **n**, Frequency and number of IFN-γ^+^TNF-α^+^ cells in tumor-infiltrating CD4^+^ T cells under the condition of LCMV GP_66–77_ stimulation. PBS (n = 5 mice), NDV-WT (n = 5 mice) and NDV-GP (n = 5 mice) in j-n. All the data are representative of at least two independent experiments. One-way ANOVA with Turkey’s test was used in b-d, f-h, j-n. Error bars (b-d, f-h, j-n) indicate s.e.m. Source data

To determine the cellular mechanism(s) underlying OV-BYTE therapy, we next analyzed the composition of important immune cells within NDV-GP-administered MC38 tumors in mice with LCMV memory (Extended Data Fig. 5a). We found that numbers of CD45^+^ immune cells were increased in tumors treated with NDV-GP compared with those of tumors treated with PBS or NDV-WT (Extended Data Fig. 5b). Moreover, the NDV-GP-mediated increased immune cell number was mainly attributed to T cells and dendritic cells (DCs) (Extended Data Fig. 5c). Further analysis of T cells revealed increased numbers of both CD8^+^ and CD4^+^ T cells in NDV-GP-treated tumors (Extended Data Fig. 5d). In addition, the ratio of CD8^+^ T cells or conventional CD4^+^ T cells to regulatory CD4^+^ T cells was largely enhanced upon NDV-GP treatment (Extended Data Fig. 5e–h). Thus, these findings suggest that NDV-GP treatment favors CD8^+^ and CD4^+^ T cell responses in the TME.

**Fig. 3:**
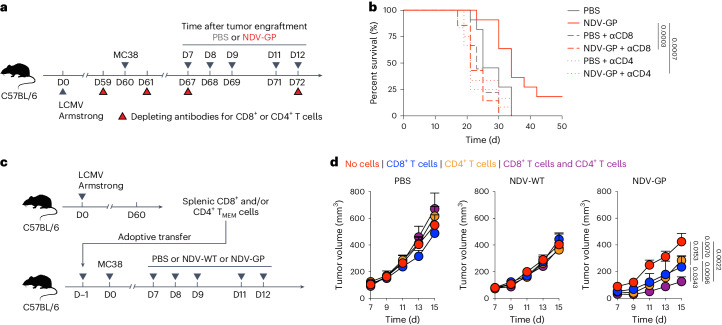
Anti-tumor effects of OV-BYTE therapy are attributed to both virus-specific CD8^+^ and CD4^+^ T_BYS_ cells. **a**, Schematic of the experimental design for **b**. Naive C57BL/6 mice were infected with LCMV Armstrong and engrafted with MC38 cells on day 60 after infection. On days 7, 8, 9, 11 and 12 after tumor engraftment, recipients were intratumorally administered PBS, NDV-WT or NDV-GP. Meanwhile, recipients were intraperitoneally injected with depleting antibodies for CD8^+^ or CD4^+^ T cells at the indicated time points. **b**, Kaplan–Meier survival curve of MC38 tumor-bearing mice intratumorally treated with PBS or NDV-GP in the presence or absence of depleting antibody for CD8^+^ (αCD8) or CD4^+^ (αCD4) T cells as described in **a**. PBS (*n* = 11 mice), NDV-GP (*n* = 11 mice), PBS and anti-CD8 antibody (*n* = 9 mice), NDV-GP and anti-CD8 antibody (*n* = 7 mice), PBS and anti-CD4 antibody (*n* = 12 mice), NDV-GP and anti-CD4 antibody (*n* = 12 mice). **c**, Schematic of the experimental design for **d**. Splenic memory CD8^+^ and/or CD4^+^ T cells were collected from LCMV Armstrong-infected mice (day 60) and adoptively transferred into naive C57BL/6 mice. These recipients were then subcutaneously engrafted with MC38 cells 1 day after adoptive cell transfer and intratumorally treated with PBS, NDV-WT or NDV-GP on days 7, 8, 9, 11 and 12 after tumor engraftment. **d**, Tumor growth curves of PBS (left; *n* = 9 (no cells), *n* = 8 (CD8^+^ T cells), *n* = 8 (CD4^+^ T cells) or *n* = 8 (CD8^+^ T cells and CD4^+^ T cells) mice), NDV-WT (middle; *n* = 9 (no cells), *n* = 8 (CD8^+^ T cells), *n* = 8 (CD4^+^ T cells) or *n* = 9 (CD8^+^ T cells and CD4^+^ T cells) mice) or NDV-GP (right; *n* = 9 (no cells), *n* = 8 (CD8^+^ T cells), *n* = 8 (CD4^+^ T cells) or *n* = 5 (CD8^+^ T cells and CD4^+^ T cells) mice) -treated MC38 tumor-bearing mice receiving adoptive transfer of LCMV Armstrong-activated CD8^+^ and/or CD4^+^ T cells or receiving no cell transfer as described in **c**. All data are representative of at least two independent experiments. The log-rank (Mantel–Cox) test was performed to compare survival curves among groups in **b**. Two-way ANOVA was used to compare tumor growth curves in **d**. Center values and error bars (**d**) indicate mean and s.e.m. Source data

To decipher the contributions of CD8^+^ and CD4^+^ T cells to the therapeutic effects of NDV-GP administration, we carried out NDV-GP treatment with CD8^+^ or CD4^+^ T cell depletion (Fig. 3a). As shown, depletion of either CD8^+^ or CD4^+^ T cells similarly abolished the therapeutic effects of NDV-GP and led to a significant reduction in long-term survival of MC38 tumor-bearing mice with LCMV memory (Fig. 3b). To further confirm the important roles of virus-specific CD8^+^ or CD4^+^ T_BYS_ cells in the OV-BYTE strategy, LCMV-specific memory CD8^+^ and/or CD4^+^ T cells were collected and adoptively transferred into naive C57BL/6 recipients, which were then engrafted with MC38 cells and intratumorally administered PBS, NDV-WT or NDV-GP (Fig. 3c). Compared to the NDV-GP-treated group with no T cell transfer, single adoptive transfer of memory CD8^+^ or CD4^+^ T cells resulted in limited tumor retardation in mice administered NDV-GP (Fig. 3d). Remarkably, the combined transfer of memory CD8^+^ and CD4^+^ T cells synergized to better control tumor growth in the NDV-GP-treated group than in monotransfer of memory CD8^+^ or CD4^+^ T cells (Fig. 3d). Nevertheless, the anti-tumor effects of LCMV-specific memory CD8^+^ and/or CD4^+^ T cell transfer were abolished upon PBS or NDV-WT treatment (Fig. 3d). Therefore, these observations indicate that OV-BYTE therapy can target virus-specific CD8^+^ and CD4^+^ T_BYS_ cells and efficiently redirect their cytotoxicity toward tumor cells.

### OV-BYTE therapy provokes cytotoxic CD4^+^ T_BYS_ cells

**Fig. 4:**
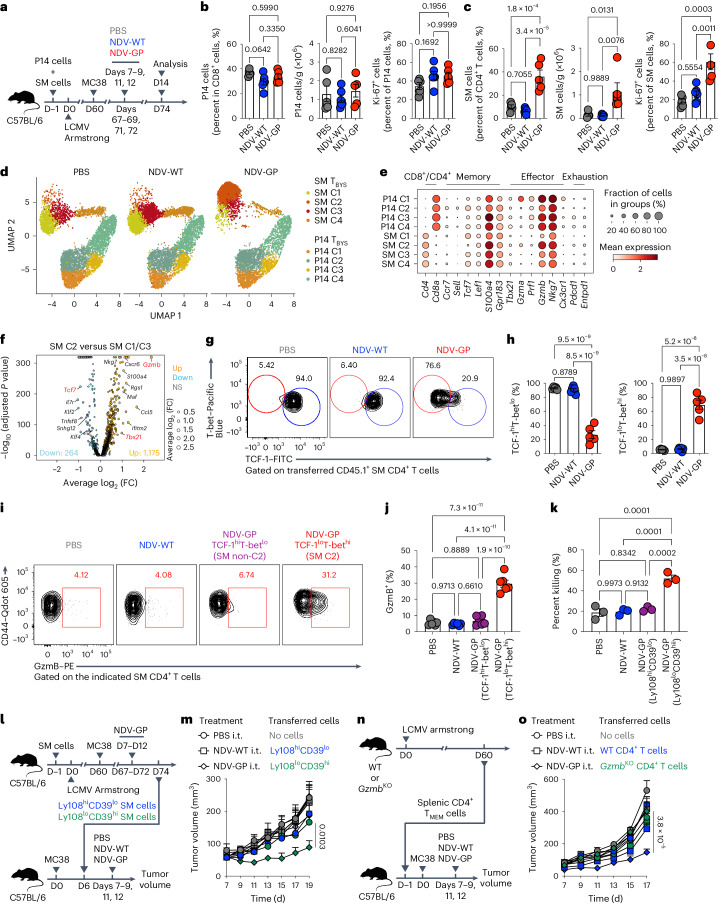
OV-BYTE therapy expands and provokes the cytotoxic effector functions of virus-specific CD4^+^ T_BYS_ cells. **a**, Schematic of the experimental design. Congenic CD45.1^+^ P14 CD8^+^ T cells and CD45.1^+^ SM CD4^+^ T cells were adoptively transferred into naive C57BL/6 recipients (CD45.2^+^), which were then infected with LCMV Armstrong and engrafted with MC38 cells on day 60 after infection. On days 7, 8, 9, 11 and 12 after tumor engraftment, recipients were intratumorally treated with PBS, NDV-WT or NDV-GP. On day 14 after tumor engraftment, tumor-infiltrating P14 and SM cells were analyzed. **b**, Frequency of P14 T_BYS_ cells of tumor-infiltrating CD8^+^ T cells (left), number of P14 T_BYS_ cells (middle) and frequency of Ki-67^+^ cells of P14 T_BYS_ cells (right) from PBS (*n* = 5 mice), NDV-WT (*n* = 6 mice) and NDV-GP (*n* = 5 mice) -treated MC38 tumors. **c**, Frequency of SM T_BYS_ cells of tumor-infiltrating CD4^+^ T cells (left), number of SM T_BYS_ cells (middle) and frequency of Ki-67^+^ cells of SM T_BYS_ cells (right) from PBS (*n* = 5 mice), NDV-WT (*n* = 6 mice) and NDV-GP (*n* = 5 mice) -treated MC38 tumors. **d**, UMAP analysis of P14 T_BYS_ and SM T_BYS_ cells upon PBS (left, 6,571 cells), NDV-WT (middle, 5,397 cells) or NDV-GP (right, 5,951 cells) treatment. **e**, Bubble chart showing selected genes in each cluster of P14 T_BYS_ and SM T_BYS_ cells as described in **d**. **f**, Volcano plot showing DEGs between SM C2 and SM C1 and SM C3 in **d**. FC, fold change; NS, not significant. **g**, Flow cytometry analysis of tumor-infiltrating SM T_BYS_ cells from the PBS-, NDV-WT- or NDV-GP-treated group. Numbers adjacent to the outlined areas indicate percentages of TCF-1^hi^T-bet^lo^ cells (blue) or TCF-1^lo^T-bet^hi^ cells (red) of SM T_BYS_ cells. **h**, Frequencies of TCF-1^hi^T-bet^lo^ cells (left) or TCF-1^lo^T-bet^hi^ cells (right) of SM T_BYS_ cells upon PBS (*n* = 5 mice), NDV-WT (*n* = 6 mice) or NDV-GP (*n* = 5 mice) treatment. **i**, Flow cytometry analysis of total SM T_BYS_ cells of the PBS- or NDV-WT-treated group and TCF-1^hi^T-bet^lo^ or TCF-1^lo^T-bet^hi^ SM T_BYS_ cells of the NDV-GP-treated group. Numbers adjacent to the outlined areas indicate percentages of GzmB^+^ cells of the indicated PBS- or NDV-WT-treated SM T_BYS_ cells or NDV-GP-treated TCF-1^hi^T-bet^lo^ or TCF-1^lo^T-bet^hi^ SM T_BYS_ cells. **j**, Frequency of GzmB^+^ cells of PBS (*n* = 6 mice) or NDV-WT (*n* = 6 mice) -treated SM T_BYS_ cells or NDV-GP-treated (*n* = 6 mice) TCF-1^hi^T-bet^lo^ or TCF-1^lo^T-bet^hi^ SM T_BYS_ cells. **k**, Ex vitro killing efficiency of SM T_BYS_ cells from the PBS (*n* = 3 mice) or NDV-WT (*n* = 3 mice) -treated group and Ly108^hi^CD39^lo^ or Ly108^lo^CD39^hi^ SM T_BYS_ cells from the NDV-GP-treated group (*n* = 3 mice). **l**, Schematic of the experimental design for **m**. Naive CD45.2^+^ C57BL/6 mice receiving adoptive transfer of congenic CD45.1^+^ SM CD4^+^ T cells were infected with LCMV Armstrong and then engrafted with MC38 cells on day 60 after infection. On days 7, 8, 9, 11 and 12 after tumor engraftment, recipients were intratumorally treated with NDV-GP. On day 14 after tumor engraftment, tumor-infiltrating Ly108^hi^CD39^lo^ or Ly108^lo^CD39^hi^ SM T_BYS_ cells were isolated and transferred into a new cohort of C57BL/6 mice, which were not exposed to LCMV Armstrong and engrafted with MC38 cells 6 days before the SM T_BYS_ cell transfer. These new recipients were next intratumorally administered PBS, NDV-WT or NDV-GP on day 7 to day 12 after tumor engraftment. **m**, Tumor growth curve of MC38 tumor-bearing mice treated with PBS, NDV-WT or NDV-GP upon Ly108^hi^CD39^lo^ (*n* = 10 (PBS), *n* = 10 (NDV-WT) or *n* = 10 (NDV-GP) mice) or Ly108^lo^CD39^hi^ (*n* = 7 (PBS), *n* = 8 (NDV-WT) or *n* = 8 (NDV-GP) mice) SM T_BYS_ cell transfer or no cell (*n* = 9 (PBS), *n* = 9 (NDV-WT) or *n* = 9 (NDV-GP) mice) transfer as described in **l**. i.t., intratumoral. **n**, Schematic of the experimental design for **o**. Naive WT C57BL/6 mice and naive *Gzmb*^KO^ mice were infected with LCMV Armstrong. On day 60 after infection, splenic CD44^+^CD127^+^ memory CD4^+^ T cells were isolated from WT or *Gzmb*^KO^ mice and then adoptively transferred into a new cohort of C57BL/6 mice. Next, these recipients were engrafted with MC38 cells and intratumorally treated with PBS, NDV-WT or NDV-GP on days 7, 8, 9, 11 and 12 after tumor inoculation. **o**, Tumor growth curve of MC38 tumor-bearing mice treated with PBS (*n* = 9 (no cells), *n* = 8 (WT CD4^+^ T cells) or *n* = 8 (*Gzmb*^KO^ CD4^+^ T cells) mice), NDV-WT (*n* = 9 (no cells), *n* = 10 (WT CD4^+^ T cells) or *n* = 9 (*Gzmb*^KO^ CD4^+^ T cells) mice) or NDV-GP (*n* = 10 (no cells), *n* = 8 (WT CD4^+^ T cells) or *n* = 10 (*Gzmb*^KO^ CD4^+^ T cells) mice) upon WT or *Gzmb*^KO^ memory CD4^+^ T cell transfer or no cell transfer as described in **n**. Data (**b**,**c**,**g**–**k**,**m**,**o**) are representative of two independent experiments. One-way ANOVA with Turkey’s test was used in **b**,**c**,**h**,**j**,**k**. The Wilcoxon signed-rank test was used in **f**. Two-way ANOVA was used to compare tumor growth curves in **m**,**o**. Center values and error bars (**b**,**c**,**h**,**j**,**k**,**m**,**o**) indicate mean and s.e.m. Source data

To ascertain how T_BYS_ cells respond to OV-BYTE therapy, C57BL/6 mice initially receiving transfer of congenic P14 and SM cells were infected with LCMV Armstrong and engrafted with MC38 cells on day 60 after infection. After five injections of PBS, NDV-WT or NDV-GP, P14 and SM T_BYS_ cells were analyzed (Fig. 4a). Notably, we found that both the frequency and number of total P14 T_BYS_ cells were comparable among PBS, NDV-WT and NDV-GP groups in the TME (Fig. 4b). Consistently, NDV-GP treatment showed no effects in boosting the proliferation of P14 T_BYS_ cells (Fig. 4b). Similarly, the frequencies and numbers of endogenous CD8^+^ T_BYS_ cells specific to LCMV GP_33–41_, GP_91–101_, GP_118–125_ and GP_276–286_ were also not influenced by NDV-GP treatment (Extended Data Fig. 5i–m). These results highlighted characteristics similar between CD8^+^ T_BYS_ cells and T_RM_ cells, both of which immediately differentiate into effector cells but with much less efficient expansion upon antigen rechallenge than T_CM_ cells^36^.

In the scenario of CD4^+^ T_BYS_ cells, we found that, in contrast to CD8^+^ T_BYS_ cells, NDV-GP treatment dramatically boosted both the frequency and number of SM T_BYS_ cells, accompanied by increased cell proliferation (Fig. 4c). Consistently, endogenous LCMV GP_66–77_-specific CD4^+^ T_BYS_ cells were also greatly expanded by NDV-GP treatment (Extended Data Fig. 5n). Thus, a biased robust increment of CD4^+^ T_BYS_ cells over CD8^+^ T_BYS_ cells is provoked by NDV-GP treatment.

**Extended Data Fig. 6:**
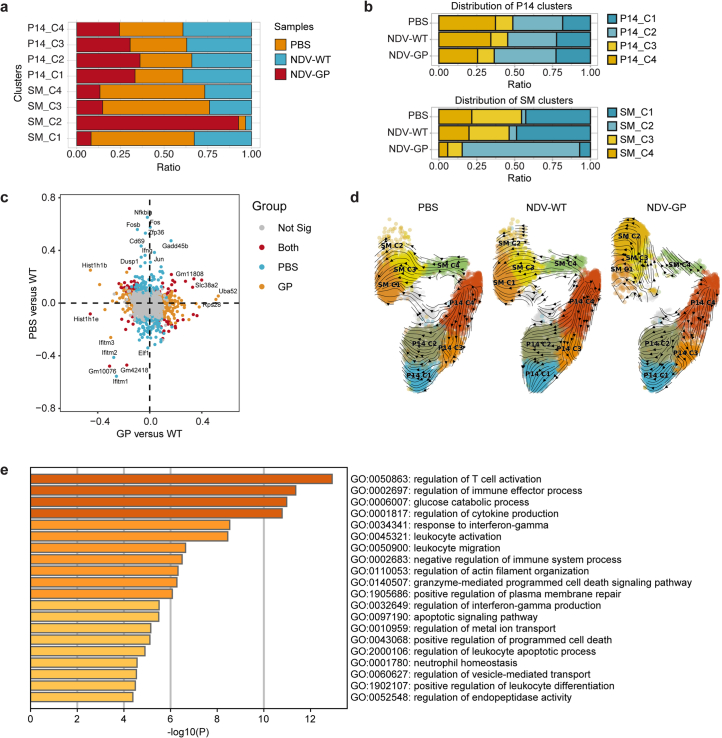
Analyses of NDV-GP-mediated T_BYS_ cell responses by scRNA-seq. **a**, Barplot showing the proportion of cells originating from PBS (6,571 cells), NDV-WT (5,397 cells) and NDV-GP (5,951 cells) groups in each cluster of P14 T_BYS_ cells (C1 to C4) or SM T_BYS_ cells (C1 to C4). **b**, Barplot showing the frequency distribution of each cluster in P14 or SM T_BYS_ cells from PBS (6,571 cells), NDV-WT (5,397 cells) or NDV-GP (5,951 cells) samples. **c**, Plot showing differential expressed genes between NDV-GP_P14 T_BYS_ cells and NDV-WT_P14 T_BYS_ cells (X-axis) and differential expressed genes between PBS_P14 T_BYS_ cells and NDV-WT_P14 T_BYS_ cells (Y-axis). **d**, Visualization of RNA velocities on diffusion map of P14 T_BYS_ cells and SM T_BYS_ cells. PBS (left, 6,571 cells), NDV-WT (middle, 5,397 cells) and NDV-GP (right, 5,951 cells). **e**, Gene Ontology analyses of up-regulated genes in SM cluster 2 as compared to SM cluster 1/3. *P* values in e were calculated based on the cumulative hypergeometric distribution. Source data

Next, we sought to delineate the molecular patterns of NDV-GP-boosted T_BYS_ cells in the TME. To this end, P14 and SM T_BYS_ cells of MC38 tumors treated with PBS, NDV-WT or NDV-GP were used for scRNA-seq analyses (Fig. 4a). A total of eight clusters were identified by uniform manifold approximation and projection (UMAP) analysis and included four clusters populated by P14 T_BYS_ cells (P14 clusters 1–4, C1–C4) and another four clusters populated by SM T_BYS_ cells (SM C1–C4) (Fig. 4d,e). Within P14 T_BYS_ cells, each cluster was evenly distributed among PBS-, NDV-WT- and NDV-GP-treated groups (Extended Data Fig. 6a,b). Moreover, we found minimally changed gene signatures in NDV-GP-treated P14 T_BYS_ cells as compared to PBS- or NDV-WT-treated ones (Extended Data Fig. 6c), suggesting that NDV-GP administration may not fundamentally alter gene signatures in P14 T_BYS_ cells of inherited T_RM_ properties^32^. By contrast, cluster composition of SM T_BYS_ cells was dramatically altered upon NDV-GP treatment, with which a pronounced enrichment of SM C2 was observed (Fig. 4d and Extended Data Fig. 6a,b). In agreement with the changed cluster composition, RNA velocity analysis further revealed a SM C2-directed differentiation in NDV-GP-educated SM T_BYS_ cells (Extended Data Fig. 6d), suggesting that SM T_BYS_ cells populating C2 might be unique responders to NDV-GP treatment.

**Extended Data Fig. 7:**
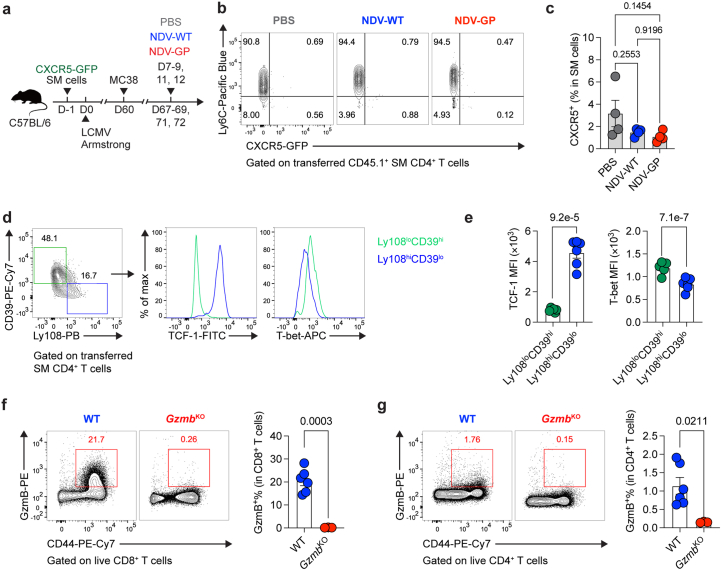
SM T_BYS_ cell differentiation upon intratumoral administration of NDV-GP. **a**, Schematic graph of the experimental design. Congenic CD45.1^+^ CXCR5-GFP SM CD4^+^ T cells were adoptively transferred into naïve C57BL/6 recipients (CD45.2^+^), which were then infected with LCMV Armstrong and engrafted with MC38 cells on day 60 after infection. On day 7, 8, 9, 11 and 12-post tumor engraftment, recipients were intratumorally treated with PBS or NDV-WT or NDV-GP. On day 14-post tumor engraftment, tumor-infiltrating CXCR5-GFP SM CD4^+^ T cells were analyzed. **b**, Flow cytometry analysis of CXCR5 and Ly6C expressions in SM T_BYS_ cells. **c**, Frequencies of CXCR5^+^ cells in SM T_BYS_ cells. PBS (n = 4 mice), NDV-WT (n = 4 mice) and NDV-GP (n = 4 mice) in c. **d**, Flow cytometry analysis of TCF-1 and T-bet expressions in Ly108^lo^CD39^hi^ and Ly108^hi^CD39^lo^ SM T_BYS_ cells from NDV-GP-treated MC38 tumors engrafted on LCMV Armstrong-infected mice. **e**, Expression levels of TCF-1 and T-bet in Ly108^lo^CD39^hi^ (n = 6 mice) and Ly108^hi^CD39^lo^ (n = 6 mice) SM T_BYS_ cells described in d. **f**, **g**, Flow cytometry analysis of GzmB-expressing CD8^+^ T cells (f) and GzmB-expressing CD4^+^ T cells (g) from the spleens of LCMV Armstrong-infected WT (n = 6 mice) and *Gzmb*^KO^ (n = 3 mice) mice on day 60^−^post infection. The numbers adjacent to the outlined areas in the left flow data panel indicate the percentages of GzmB-expressing cells in CD8^+^ T cells (f) or CD4^+^ T cells (g), which are summarized on the right panel. All data are representative of at least two independent experiments. One-way ANOVA with Turkey’s test was used in c. Two-tailed unpaired Student’s *t*-test was used in e-g. Error bars (c, e-g) indicate s.e.m. Source data

To characterize the molecular signature and functions of NDV-GP-educated SM T_BYS_ cells, we further determined differentially expressed genes (DEGs) between SM C2 and other SM clusters. A total of 1,175 and 264 DEGs were upregulated and downregulated, respectively, in C2 of SM T_BYS_ cells. Specifically, expression of genes involved in cytotoxic functions (for example, *Gzmb*, *Ccl5*, *Tbx21*) was largely enhanced in SM C2, while expression of genes regulating T cell memory such as *Tcf7*, *Il7r* and *Klf2* was decreased (Fig. 4f). Further gene ontology analysis of SM C2 showed significant enrichment of terms related to regulation of T cell activation and regulation of the immune effector process (Extended Data Fig. 6e), indicative of active effector functions of NDV-GP-educated SM T_BYS_ C2 cells. Furthermore, by virtue of flow cytometry, we confirmed the presence of NDV-GP treatment-induced SM C2 cells as the TCF-1^lo^T-bet^hi^ SM T_BYS_ subset (Fig. 4g,h), highly expressing granzyme B (GzmB), which was consistent with transcription profiles of SM C2 cells (Fig. 4i,j). Moreover, NDV-GP-educated SM T_BYS_ cells still preserved T_H_1 cell identity as indicated by the abundance of Ly6C and the absence of CXCR5 (Extended Data Fig. 7a–c). These findings suggest that a noticeable memory-to-effector transition is involved in the CD4^+^ T_BYS_ cell response to the OV carrying cognate epitopes.

To further investigate the potential cytotoxic functions of NDV-GP-educated TCF-1^lo^T-bet^hi^CD4^+^ T_BYS_ cells, we performed ex vivo killing assays with TCF-1^lo^T-bet^hi^ and TCF-1^hi^T-bet^lo^ SM T_BYS_ cells from the NDV-GP-treated group as well as SM T_BYS_ cells from PBS- or NDV-WT-treated groups. Here, Ly108 and CD39 were used as surrogate surface markers to distinguish and isolate TCF-1^lo^T-bet^hi^ (Ly108^lo^CD39^hi^) and TCF-1^hi^T-bet^lo^ (Ly108^hi^CD39^lo^) SM T_BYS_ cells (Extended Data Fig. 7d,e). We found that NDV-GP-educated TCF-1^lo^T-bet^hi^ (Ly108^lo^CD39^hi^) SM T_BYS_ cells were more efficient at directly killing target cells than TCF-1^hi^T-bet^lo^ (Ly108^hi^CD39^lo^) SM T_BYS_ cells from the NDV-GP group and other control SM T_BYS_ cells from the PBS or NDV-WT group (Fig. 4k), indicating superior cytotoxic functions of NDV-GP-educated TCF-1^lo^T-bet^hi^ SM T_BYS_ cells. To more directly evaluate the reliance on TCF-1^lo^T-bet^hi^CD4^+^ T_BYS_ cells for the therapeutic effects of the OV-BYTE strategy, TCF-1^lo^T-bet^hi^ (Ly108^lo^CD39^hi^) or TCF-1^hi^T-bet^lo^ (Ly108^hi^CD39^lo^) SM T_BYS_ cells from NDV-GP-administered MC38 tumors were adoptively transferred into MC38 tumor-engrafted recipients with no exposure to LCMV Armstrong infection, and then these recipients were intratumorally administered PBS, NDV-WT or NDV-GP (Fig. 4l). Indeed, only transfer of the TCF-1^lo^T-bet^hi^ SM T_BYS_ subset, but not transfer of TCF-1^hi^T-bet^lo^ SM T_BYS_ cells, could mediate the anti-tumor effect of NDV-GP treatment (Fig. 4m).

It is well known that GzmB is important for the cytotoxicity of cytotoxic T cells^37^. Here, we also found that GzmB was specifically expressed in NDV-GP-induced cytotoxic TCF-1^lo^T-bet^hi^ T_BYS_ cells (Fig. 4f,i,j); however, whether GzmB expression is a prerequisite for the cytotoxicity of these TCF-1^lo^T-bet^hi^ T_BYS_ cells remains unknown. To this end, LCMV-specific memory CD4^+^ T cells were isolated from WT C57BL/6 mice or GzmB-deficient (*Gzmb*^KO^) mice on day 60 after infection and then transferred into naive C57BL/6 recipients as CD4^+^ T_BYS_ cells. These recipients were then engrafted with MC38 cells and intratumorally treated with PBS, NDV-WT or NDV-GP (Fig. 4n and Extended Data Fig. 7f,g). Consistently, transfer of WT LCMV-specific memory CD4^+^ T cells potentiated the anti-tumor function of NDV-GP administration in recipients without LCMV Armstrong exposure. Nevertheless, such protection was completely abolished in the absence of GzmB expression in CD4^+^ T cells (Fig. 4o). In aggregate, these results highlight the GzmB-dependent cytotoxicity of CD4^+^ T_BYS_ cells as indispensable in achieving therapeutic outcome of OV-BYTE therapy.

### OV-BYTE promotes in situ T_BYS_ cells to restrain tumor growth

**Fig. 5:**
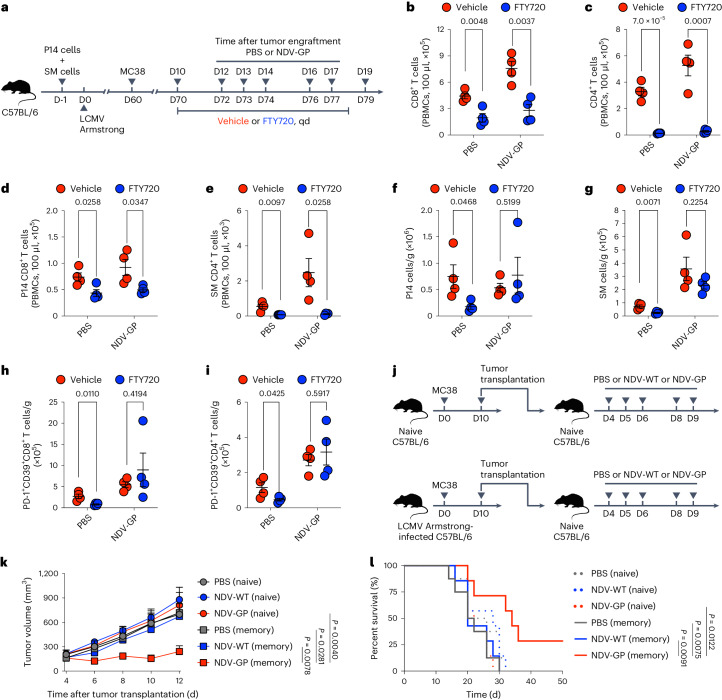
T_BYS_ cells bolstered in situ by OV-BYTE therapy contribute to tumor control. **a**, Schematic of the experimental design. Congenic CD45.1^+^ P14 CD8^+^ T cells and CD45.1^+^ SM CD4^+^ T cells were adoptively transferred into naive C57BL/6 recipients, which were then infected with LCMV Armstrong and engrafted with MC38 cells on day 60 after infection. On days 12, 13, 14, 16 and 17 after tumor engraftment, recipients were intratumorally treated with PBS or NDV-GP. Meanwhile, the recipients were gavaged with FTY720 drug or vehicle from day 10 to day 18. On day 19 after tumor engraftment, tumor-infiltrating T cells were analyzed. qd, quaque die. **b**–**e**, Numbers of CD8^+^ T cells (**b**), CD4^+^ T cells (**c**), P14 cells (**d**) and SM cells (**e**) in the peripheral blood of mice in each group as described in **a**. **f**,**g**, Numbers of tumor-infiltrating P14 T_BYS_ cells (**f**) and SM T_BYS_ cells (**g**). **h**,**i**, Numbers of tumor-infiltrating PD-1^+^CD39^+^CD8^+^ T cells (**h**) and PD-1^+^CD39^+^CD4^+^ T cells (**i**). PBS and vehicle (*n* = 4 mice), PBS and FTY720 (*n* = 4 mice), NDV-GP and vehicle (*n* = 4 mice) and NDV-GP and FTY720 (*n* = 4 mice) in **b**–**i**. **j**, Schematic of the experimental design for **k**,**l**. LCMV Armstrong-infected (day 60) or naive (uninfected) C57BL/6 mice were engrafted with MC38 cells. On day 10 after tumor engraftment, MC38 tumors were dissected from infected or uninfected mice and transplanted to naive C57BL/6 mice, which were then intratumorally treated with PBS, NDV-WT or NDV-GP on days 4, 5, 6, 8 and 9 after tumor transplantation. **k**,**l**, Tumor growth curve (**k**) and Kaplan–Meier survival curve (**l**) of MC38 tumor mass-recipient mice treated with PBS (*n* = 5 (naive in **k**), *n* = 8 (naive in **l**), *n* = 8 (memory in **k**) or *n* = 8 (memory in **l**) mice), NDV-WT (*n* = 5 (naive in **k**), *n* = 8 (naive in **l**), *n* = 7 (memory in **k**) or *n* = 7 (memory in **l**) mice) or NDV-GP (*n* = 7 (naive in **k**), *n* = 7 (naive in **l**), *n* = 6 (memory in **k**) or *n* = 7 (memory in **l**) mice) as described in **j**. ‘Naive’ indicates mice transplanted with MC38 tumors originating from uninfected mice; ‘memory’ indicates mice transplanted with MC38 tumors originating from LCMV Armstrong-infected mice. All data are representative of two independent experiments. Two-tailed unpaired Student’s *t*-test was used in **b**–**i**. The log-rank (Mantel–Cox) test was performed to compare survival curves among groups in **k**. Two-way ANOVA was used to compare tumor growth curves in **l**. Center values and error bars (**b**–**i**,**k**) indicate mean and s.e.m. Source data

Reportedly, T_TST_ cells in the tumor-draining lymph node (dLN) serve as a reservoir for their counterparts in the TME that are maintained by continuous migration^19,38^, which calls into question whether OV-BYTE relies on dLN egressing or TME-resident T_BYS_ cells for its anti-tumor efficacy. To this end, naive C57BL/6 mice with adoptive transfer of P14 and SM cells were infected with LCMV Armstrong and then engrafted with MC38 cells on day 60 after infection. On day 10 after tumor incubation, mice were gavaged with the sphingosine-1-phosphate receptor agonist FTY720 (ref. ^39^) to block dLN egress and then intratumorally administered PBS or NDV-GP. As a control, MC38-bearing mice gavaged with PBS vehicle were intratumorally administered PBS or NDV-GP (Fig. 5a). As shown, FTY720 treatment blunted the numbers of CD8^+^ T cells, CD4^+^ T cells, P14 cells and SM cells in the peripheral blood of mice intratumorally administered PBS or NDV-GP (Fig. 5b–e). In addition, FTY720 treatment yielded reductions of both P14 and SM T_BYS_ cells in mice intratumorally administered PBS (Fig. 5f,g), suggesting that the migration of T_MEM_ cells from dLNs is likely necessary to sustain T_BYS_ cells in the TME. Remarkably, we observed that the FTY720-mediated decrease of P14 and SM T_BYS_ cells in the TME was almost completely rescued by intratumoral administration of NDV-GP (Fig. 5f,g), indicating that NDV-GP treatment can efficiently expand in situ virus-specific T_BYS_ cells even if the replenishment of these cells from dLNs were disrupted. Importantly, we further observed that the FTY720-mediated decrease in PD-1^+^CD39^+^ T cells^12,40^ was also rectified by NDV-GP treatment (Fig. 5h,i), which might be due to enhanced antigen presentation upon NDV-GP treatment (Extended Data Fig. 4e,f) that optimizes T cell responses in the TME.

To further interrogate whether the in situ amplified T_BYS_ cells were responsible for OV-BYTE therapeutic efficacy, naive and LCMV Armstrong-infected C57BL/6 mice were engrafted with MC38 cells. On day 10 after tumor engraftment, MC38 tumor masses were dissected from naive or infected mice and subcutaneously transplanted to naive C57BL/6 recipients, which were then intratumorally administered PBS, NDV-WT or NDV-GP (Fig. 5j). We found that NDV-GP administration better controlled the growth of transplanted MC38 tumors with pre-existing LCMV GP-specific T cells in the tumor mass and prolonged the survival of mice transplanted with such tumors (Fig. 5k,l), mirroring the long-term protection afforded by NDV-GP in MC38 tumor-engrafted mice with LCMV Armstrong infection (Fig. 2a–c). Hence, in situ amplified virus-specific T_BYS_ cells primarily contribute to the therapeutic effects of OV-BYTE therapy.

### OV-BYTE augments T_TST_ cells by inducing epitope spreading

**Fig. 6:**
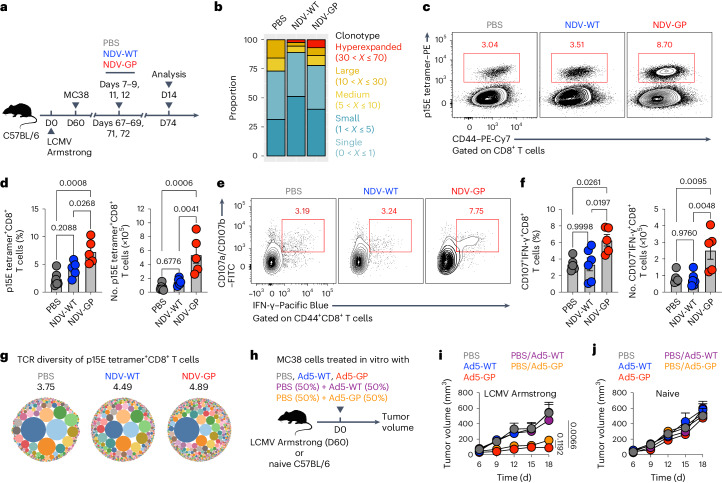
OV-BYTE therapy triggers T_TST_ cell responses by inducing T cell epitope spreading. **a**, Schematic of the experimental design. Naive C57BL/6 mice were infected with LCMV Armstrong and engrafted with MC38 cells on day 60 after infection. On days 7, 8, 9, 11 and 12 after tumor engraftment, MC38 tumor-engrafted mice were intratumorally treated with PBS, NDV-WT or NDV-GP. On day 14 after tumor engraftment, tumor-infiltrating T cell responses were analyzed. **b**, Frequencies of TCR clonotypes in PD-1^+^CD39^+^ tumor-reactive CD8^+^ T cells originating from PBS (*n* = 3,222 cells), NDV-WT (*n* = 966 cells) or NDV-GP (*n* = 1,565 cells) -treated MC38 tumors on day 14 after tumor engraftment as indicated in **a**. These TCR clonotypes were divided into five groups of different sizes, including hyperexpanded (30 < *X* ≤ 70), large (10 < *X* ≤ 30), medium (5 < *X* ≤ 10), small (1 < *X* ≤ 5) and single (0 < *X* ≤ 1). **c**, Flow cytometry analysis of MC38 tumor-infiltrating CD44^+^CD8^+^ T cells from the PBS-, NDV-WT- or NDV-GP-treated group on day 14 after tumor engraftment as indicated in **a**. Numbers adjacent to the outlined areas indicate percentages of p15E tetramer^+^CD44^hi^ tumor-specific CD8^+^ T cells of tumor-infiltrating CD8^+^ T cells. **d**, Frequency and number of tumor-infiltrating p15E tetramer^+^CD44^hi^ tumor-specific CD8^+^ T cells in **c**. PBS (*n* = 6 mice), NDV-WT (*n* = 5 mice) and NDV-GP (*n* = 5 mice). **e**, Flow cytometry analysis of tumor-infiltrating CD44^+^CD8^+^ T cells (under the condition of p15E peptide stimulation) from the PBS-, NDV-WT- or NDV-GP-treated group on day 14 after tumor engraftment as indicated in **a**. Numbers adjacent to the outlined areas indicate frequencies of IFN-γ^+^CD107^+^ cells of tumor-infiltrating CD44^+^CD8^+^ T cells. **f**, Frequency and number of tumor-infiltrating p15E peptide-stimulated IFN-γ^+^CD107^+^CD8^+^ T cells in **e**. PBS (*n* = 5 mice), NDV-WT (*n* = 6 mice) and NDV-GP (*n* = 5 mice). **g**, Bubble charts showing Shannon entropy analysis of TCR diversity in tumor-infiltrating p15E tetramer^+^CD39^hi^CD8^+^ T cells from the PBS-, NDV-WT- or NDV-GP-treated group on day 14 after tumor engraftment as indicated in **a**. **h**, Schematic of the experimental design. MC38 cells were treated in vitro with PBS, Ad5-WT (multiplicity of infection of 1) or Ad5-GP (multiplicity of infection of 1) for 48 h. Next, naive or LCMV Armstrong-infected C57BL/6 mice were inoculated with the same amount of PBS-, Ad5-WT- or Ad5-GP-treated MC38 cells or a 50:50 mix of PBS- and Ad5-GP-treated MC38 cells or a 50:50 mix of PBS- and Ad5-WT-treated MC38 cells. **i**,**j**, Tumor growth curves of the MC38 cells described in **h** engrafted in LCMV Armstrong-infected C57BL/6 mice (**i**) or naive C57BL/6 mice (**j**). PBS (*n* = 6 mice), Ad5-WT (*n* = 6 mice), Ad5-GP (*n* = 4 mice), PBS and Ad5-WT (*n* = 5 mice) and PBS and Ad5-GP (*n* = 4 mice) in **i**; PBS (*n* = 6 mice), Ad5-WT (*n* = 6 mice), Ad5-GP (*n* = 5 mice), PBS and Ad5-WT (*n* = 4 mice) and PBS and Ad5-GP (*n* = 6 mice) in **j**. Data (**c**–**f**,**i**,**j**) are representative of two independent experiments. One-way ANOVA with Turkey’s test was used in **d**,**f**. Two-way ANOVA was used to compare tumor growth curves in **i**,**j**. Center values and error bars (**d**,**f**,**i**,**j**) indicate mean and s.e.m. Source data

Next, we aimed to evaluate intrinsic T_TST_ cell responses upon OV-BYTE therapy. To this end, we first investigated changes of the T cell receptor (TCR) repertoire in PD-1^+^CD39^+^CD8^+^ T cells originating from PBS-, NDV-WT- or NDV-GP-treated MC38 tumors engrafted on LCMV-infected mice by performing single-cell TCR sequencing (scTCR-seq) analysis (Fig. 6a). The results revealed enhanced oligoclonal expansion of tumor-reactive CD8^+^ T cells of the NDV-GP group in relation to those of the PBS or NDV-WT groups (Fig. 6b), suggesting potential T cell epitope spreading of tumor-reactive CD8^+^ T cells in the presence of OV-BYTE therapy. Further examination of endogenous CD8^+^ T cells specific to the MC38 tumor antigen p15E by both tetramer staining and a relevant peptide stimulation assay highlighted the reinforced p15E-specific CD8^+^ T cell response, including both quantity and quality, upon NDV-GP treatment (Fig. 6c–f). Moreover, in-depth TCR repertoire analysis indicated that p15E-specific CD8^+^ T cells from the NDV-GP group exhibited TCR clones of greater breadth and diversity than their counterparts from PBS or NDV-WT groups (Fig. 6g), further corroborating the notion that OV-BYTE therapy targeting virus-specific epitopes virtually led to epitope spreading of tumor-specific antigens and, as a result, enhanced T_TST_ cell responses.

For further proof of the anti-tumor effects caused by OV-BYTE-driven TCR epitope spreading, we inoculated C57BL/6 naive mice or those with LCMV memory with a 50:50 mix of PBS-treated and Ad5-GP-treated MC38 cells. As controls, C57BL/6 naive mice or those with LCMV memory were inoculated with the same amount of PBS-, Ad5-WT- or Ad5-GP-treated MC38 cells as well as a 50:50 mix of PBS- and Ad5-WT-treated ones (Fig. 6h). It transpired that the tumor growth of Ad5-GP-treated MC38 cells was significantly inhibited in mice with LCMV memory but not in naive mice. Also, such tumor growth inhibition was also preserved in the setting of the mixed PBS- and Ad5-GP-treated MC38 cells engrafted in mice with LCMV memory (Fig. 6i,j), indicating the development of anti-tumor responses against tumor cells expressing no viral antigens upon OV-BYTE therapy.

**Extended Data Fig. 8:**
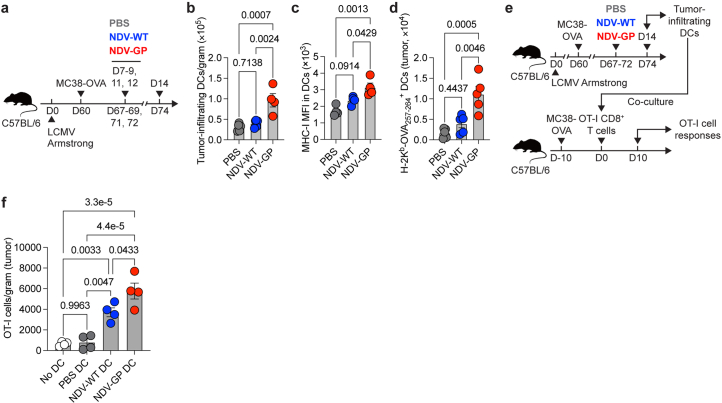
DCs contribute to the OV-BYTE therapy-induced T cell epitope spreading. **a**, Schematic graph of the experimental design. Naïve C57BL/6 mice were infected with LCMV Armstrong and engrafted with MC38-OVA cells on day 60 after infection. These tumor-engrafted mice were then daily treated with PBS or NDV-WT or NDV-GP on day 7, 8, 9, 11 and 12 after tumor inoculation. On day 14, tumor-infiltrating immune cells were analyzed. **b**, Number of CD45^+^F4/80^−^CD11c^+^MHC-II^+^ DCs originating from PBS- (n = 5 mice) or NDV-WT- (n = 5 mice) or NDV-GP-treated (n = 4 mice) MC38-OVA tumors described in a. **c**, MHC-I expression levels in CD45^+^F4/80^-^CD11c^+^MHC-II^+^ DCs originating from PBS- (n = 4 mice) or NDV-WT- (n = 4 mice) or NDV-GP-treated (n = 4 mice) MC38-OVA tumors described in a. **d**, Number of CD45^+^F4/80^-^CD11c^+^I-A^b+^H-2K^b^-OVA_257–264_^+^ DCs originating from PBS- (n = 5 mice) or NDV-WT- (n = 5 mice) or NDV-GP-treated (n = 5 mice) MC38-OVA tumors described in a. **e**, Schematic graph of the experimental design. Naïve C57BL/6 mice were infected with LCMV Armstrong and engrafted with MC38-OVA cells on day 60 after infection. These mice were then treated with PBS or NDV-WT or NDV-GP from day 7 to day 12 after tumor inoculation. On day 14, tumor-infiltrating DCs from PBS or NDV-WT or NDV-GP groups were isolated, labeled with OVA_257–264_ peptide and cultured with naïve OT-I CD8^+^ T cells overnight. The next day, OT-I CD8^+^ T cells were transferred into a new cohort of MC38-OVA-engrafted mice and analyzed on day 10-post transfer. **f**, Numbers of MC38-OVA tumor-infiltrating OT-I CD8^+^ T cells pre-cultured with DCs from PBS (n = 4 mice) or NDV-WT (n = 4 mice) or NDV-GP (n = 4 mice) groups and OT-I CD8^+^ T cells without DC co-culture (n = 4 mice) as described in e. All the data are representative of two independent experiments. One-way ANOVA with Turkey’s test was used in b-d, f. Error bars (b-d, f) indicate s.e.m. Source data

To further explore the potential mechanism(s) underlying OV-BYTE therapy-driven TCR epitope spreading, mice with LCMV memory were engrafted with MC38 cells expressing ovalbumin (OVA) (hereafter referred to as MC38-OVA) and were then administered PBS, NDV-WT or NDV-GP intratumorally (Extended Data Fig. 8a). Given the amenable role of DCs in fostering TCR epitope spreading of tumor-specific T cells^41,42^, we next examined the DC response in the aforementioned experimental settings, in which we observed an enhanced DC response in NDV-GP-treated MC38-OVA tumors as compared to that in PBS- or NDV-WT-treated ones (Extended Data Fig. 8b,c). More specifically, we further accessed the presentation of OVA_257–264_ peptide by MHC-I H-2K^b^ in DCs and found that DCs presenting OVA_257–264_ peptide were increased in the NDV-GP group as compared to those in the PBS or NDV-WT group (Extended Data Fig. 8d). In addition, DCs isolated from NDV-GP-treated MC38-OVA tumors rather than PBS- or NDV-WT-treated ones were more efficient in priming the tumor-specific OT-I CD8^+^ T cell response (Extended Data Fig. 8e,f). In sum, these data provide evidence that OV-BYTE therapy targeting bystander viral epitopes may license DCs to enhance the presentation of spreading tumor-specific epitopes to CD8^+^ T_TST_ cells.

### OV-BYTE and PD-L1 ICB synergize to control tumor progression

**Fig. 7:**
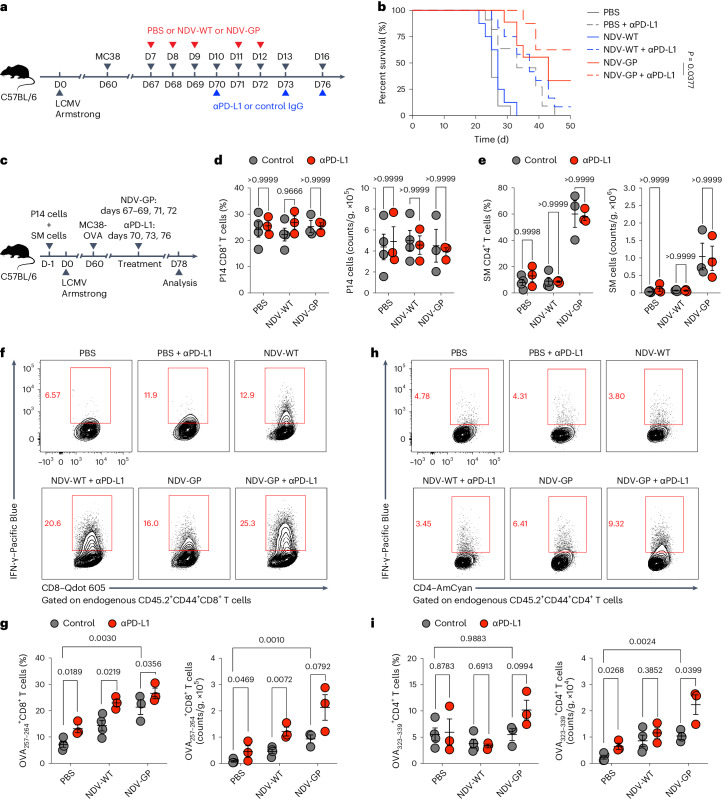
OV-BYTE therapy and PD-L1 ICB therapy synergize to control tumor progression. **a**, Schematic of the experimental design for **b**. Naive C57BL/6 mice were infected with LCMV Armstrong and engrafted with MC38 cells on day 60 after infection. On days 7, 8, 9, 11 and 12 after tumor engraftment, recipients were intratumorally administered PBS, NDV-WT or NDV-GP. Meanwhile, recipients of each group were administered anti-PD-L1 blocking antibody (αPD-L1) or control immunoglobulin G (IgG) antibody on days 10, 13 and 16 after tumor engraftment. **b**, Kaplan–Meier survival curve for **a**. PBS and control (*n* = 11 mice), PBS and anti-PD-L1 antibody (*n* = 11 mice), NDV-WT and control (*n* = 8 mice), NDV-WT and anti-PD-L1 antibody (*n* = 12 mice), NDV-GP and control (*n* = 9 mice) and NDV-GP and anti-PD-L1 antibody (*n* = 8 mice). **c**, Schematic of the experimental design. Congenic CD45.1^+^ P14 CD8^+^ T cells and CD45.1^+^ SM CD4^+^ T cells were adoptively transferred into naive C57BL/6 recipients, which were then infected with LCMV Armstrong and engrafted with MC38-OVA cells on day 60 after infection. On days 7, 8, 9, 11 and 12 after tumor engraftment, recipients were intratumorally treated with PBS, NDV-WT or NDV-GP. Meanwhile, recipients were treated with anti-PD-L1 antibody or control IgG antibody on days 10, 13 and 16 after tumor engraftment. On day 18 after tumor engraftment, tumor-infiltrating T cells were analyzed. **d**,**e**, Frequencies and numbers of P14 T_BYS_ cells of tumor-infiltrating CD8^+^ T cells (**d**) and SM T_BYS_ cells of tumor-infiltrating CD4^+^ T cells (**e**) from each group described in **c**. PBS and control (*n* = 4 mice), PBS and anti-PD-L1 antibody (*n* = 3 mice), NDV-WT and control (*n* = 4 mice), NDV-WT and anti-PD-L1 antibody (*n* = 3 mice), NDV-GP and control (*n* = 3 mice), NDV-GP and anti-PD-L1 antibody (*n* = 3 mice) in **d**,**e**. **f**, Flow cytometry analysis of tumor-infiltrating endogenous CD45.2^+^CD44^+^CD8^+^ T cells. Numbers adjacent to outlined areas indicate percentages of IFN-γ^+^ cells of CD45.2^+^CD44^+^CD8^+^ T cells under the condition of ex vivo OVA_257–264_ stimulation. **g**, Frequencies and numbers of OVA_257–264_-stimulated IFN-γ^+^CD8^+^ T cells. PBS and control (*n* = 4 mice), PBS and anti-PD-L1 antibody (*n* = 3 mice), NDV-WT and control (*n* = 4 mice), NDV-WT and anti-PD-L1 antibody (*n* = 3 mice), NDV-GP and control (*n* = 3 mice), NDV-GP and anti-PD-L1 antibody (*n* = 3 mice) in **g**. **h**, Flow cytometry analysis of tumor-infiltrating endogenous CD45.2^+^CD44^+^CD4^+^ T cells. Numbers adjacent to outlined areas indicate percentages of IFN-γ^+^ cells of CD45.2^+^CD44^+^CD4^+^ T cells under the condition of ex vivo OVA_323–339_ stimulation. **i**, Frequencies and numbers of OVA_323–339_-stimulated IFN-γ^+^CD4^+^ T cells. PBS and control (*n* = 4 mice), PBS and anti-PD-L1 antibody (*n* = 3 mice), NDV-WT and control (*n* = 4 mice), NDV-WT and anti-PD-L1 antibody (*n* = 3 mice), NDV-GP and control (*n* = 3 mice), NDV-GP and anti-PD-L1 antibody (*n* = 3 mice) in **i**. All data are representative of two independent experiments. The log-rank (Mantel–Cox) test was performed to compare survival curves among groups in **b**. Two-tailed unpaired Student’s *t*-test was used in **d**,**e**,**g**,**i**. Center value and error bars (**d**,**e**,**g**,**i**) indicate mean and s.e.m. Source data

Distinct from OV-BYTE therapy that targets T_BYS_ cells, PD-1 and/or PD-L1 ICB therapy is suggested to reinvigorate T_EX_ or T_TST_ cells^2^. As effective OV treatment has been reported to remodel the TME to favor PD-1 and/or PD-L1 ICB therapy^43^, we hypothesized that OV-BYTE therapy would show a synergistic anti-tumor response with PD-1 and/or PD-L1 ICB therapy. Indeed, C57BL/6 mice with LCMV memory concurrently treated with NDV-GP and PD-L1 ICB demonstrated ~60% complete protection against MC38 tumor challenge, which was superior to treatment with NDV-GP or PD-L1 ICB therapy alone (Fig. 7a,b). To further understand how T_BYS_ and T_TST_ cells respond to the combined NDV-GP and PD-L1 ICB therapy, C57BL/6 mice with LCMV memory and adoptively transferred P14 and SM cells were engrafted with MC38-OVA cells and then administered NDV-GP and PD-L1 ICB therapy (Fig. 7c). Consistent with the aforementioned data, P14 T_BYS_ cells in MC38-OVA tumors were comparable among PBS, NDV-WT and NDV-GP groups (Fig. 7d). Meanwhile, the SM T_BYS_ cell response of NDV-GP treatment was bolstered compared to that of PBS or NDV-WT treatment (Fig. 7e). However, P14 and SM T_BYS_ cells, both of which barely expressed PD-1 (Fig. 1d,j), were minimally influenced by PD-L1 ICB therapy in all groups (Fig. 7d,e). We then examined T_TST_ cell responses by ex vivo OVA_257–264_ (CD8^+^ T cell epitope) or OVA_323–339_ (CD4^+^ T cell epitope) peptide stimulation assays. Notably, the OVA_257–264_-specific CD8^+^ T cell response was most enhanced by concurrent treatment with PD-L1 ICB and NDV-GP in all experimental settings, evidenced by the highest proportion and number of IFN-γ-producing CD8^+^ T cells upon OVA_257–264_ stimulation (Fig. 7f,g). Similarly, the OVA_323–339_-specific CD4^+^ T cell response in the NDV-GP and PD-L1 ICB combinatorial treatment group was also superior to that of all other groups (Fig. 7h,i). Collectively, these findings demarcate that OV-BYTE potentiates the anti-tumor efficacy of PD-L1 ICB by augmenting T_TST_ cell responses.

### OV-BYTE enables anti-tumoral SARS-CoV-2-specific T_MEM_ cells

**Fig. 8:**
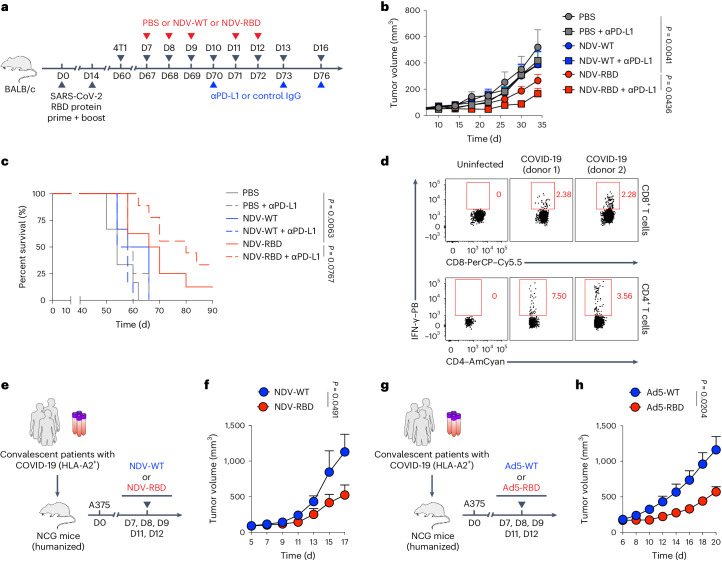
OV-BYTE therapy potentiates anti-tumor activities of SARS-CoV-2-specific T_MEM_ cells. **a**, Schematic of the experimental design for **b**. Naive BALB/c mice were prime–boost immunized with SARS-CoV-2 RBD protein at an interval of 14 days and engrafted with 4T1 cells on day 60 after prime immunization. On days 7, 8, 9, 11 and 12 after tumor engraftment, recipients were intratumorally administered PBS, NDV-WT or NDV-RBD. Meanwhile, recipients of each group were administered anti-PD-L1 blocking antibody or control IgG antibody on days 10, 13 and 16 after tumor engraftment. **b**,**c**, Tumor growth curve (**b**) and Kaplan–Meier survival curve (**c**) from **a**. PBS and control (*n* = 7 mice), PBS and anti-PD-L1 antibody (*n* = 6 mice), NDV-WT and control (*n* = 5 mice), NDV-WT and anti-PD-L1 antibody (*n* = 6 mice), NDV-RBD and control (*n* = 7 mice) and NDV-RBD and anti-PD-L1 antibody (*n* = 7 mice) in **b**. PBS and control (*n* = 6 mice), PBS and anti-PD-L1 antibody (*n* = 4 mice), NDV-WT and control (*n* = 6 mice), NDV-WT and anti-PD-L1 antibody (*n* = 6 mice), NDV-RBD and control (*n* = 8 mice) and NDV-RBD and anti-PD-L1 antibody (*n* = 9 mice) in **c**. **d**, Flow cytometry analysis of CD8^+^ (top) and CD4^+^ (bottom) T cells from the PBMCs of SARS-CoV-2-uninfected donors and convalescent donor with COVID-19. Numbers adjacent to the outlined areas indicate percentages of SARS-CoV-2 RBD-stimulated IFN-γ^+^ cells of CD8^+^ T cells or IFN-γ^+^ cells of CD4^+^ T cells. **e**, Schematic experimental design for **f**. NCG mice were engrafted with PBMCs from HLA-A2-positive convalescent patients with COVID-19. After reconstruction of human T cells, these humanized NCG mice were engrafted with A375 cells and then intratumorally administered NDV-WT or NDV-RBD on days 7, 8, 9, 11 and 12 after tumor engraftment. **f**, Tumor growth curve of A375 tumor-bearing humanized NCG mice intratumorally treated with NDV-WT (*n* = 5 mice) or NDV-RBD (*n* = 4 mice) as indicated in **e**. **g**, Schematic experimental design for **h**. NCG mice were engrafted with PBMCs from HLA-A2-positive convalescent patients with COVID-19. Next, these reconstructed humanized NCG mice were engrafted with A375 cells and then intratumorally administered Ad5-WT or Ad5-RBD on days 7, 8, 9, 11 and 12 after tumor engraftment. **h**, Tumor growth curve of A375 tumor-bearing humanized NCG mice intratumorally treated with Ad5-WT (*n* = 6 mice) or Ad5-RBD (*n* = 6 mice) as indicated in **g**. Data (**b**–**d**,**f**,**h**) are representative of two independent experiments. Two-way ANOVA was used to compare tumor growth curves in **b**,**f**,**h**. The log-rank (Mantel–Cox) test was performed to compare survival curves among groups in **c**. Center values and error bars (**b**,**f**,**h**) indicate mean and s.e.m. Source data

**Extended Data Fig. 9:**
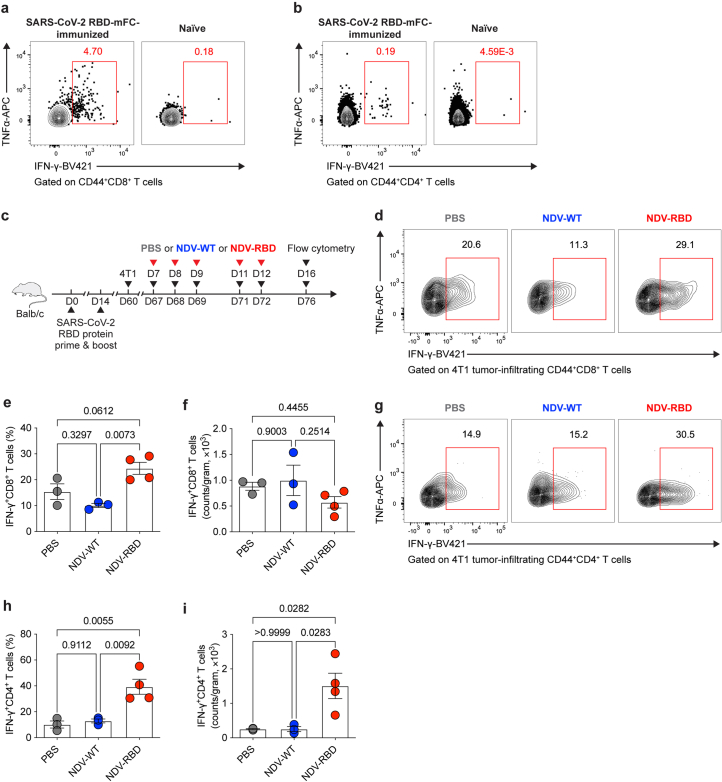
SARS-CoV-2 RBD-specific T_BYS_ cell responses to NDV-RBD. **a, b**, Flow cytometry analysis of splenic CD8^+^ T cells (a) and CD4^+^ T cells (b) under the condition of SARS-CoV-2 protein stimulation. The numbers adjacent to the outlined areas indicate the frequencies of IFN-γ^+^ cells in CD44^+^CD8^+^ T cells (a) or CD44^+^CD4^+^ T cells (b). **c**, Schematic graph of the experimental design. Naïve Balb/c mice were immunized with SARS-CoV-2 RBD protein and engrafted with 4T1 cells on day 60 after immunization. On day 7, 8, 9, 11 and 12 after tumor engraftment, recipients were intratumorally administrated with PBS or NDV-WT or NDV-RBD. On day 16 after tumor engraftment, tumor-infiltrating T cells were analyzed. **d**, Flow cytometry analysis of 4T1 tumor-infiltrating CD44^+^CD8^+^ T cells under the condition of SARS-CoV-2 S1 peptide pool stimulation. The numbers adjacent to the outlined areas indicate the frequencies of IFN-γ^+^ cells in CD44^+^CD8^+^ T cells. **e, f**, Frequency (e) and number (f) of SARS-CoV-2 S1-stimulated IFN-γ^+^CD8^+^ T cells in d. **g**, Flow cytometry analysis of 4T1 tumor-infiltrating CD44^+^CD4^+^ T cells under the condition of SARS-CoV-2 S1 peptide pool stimulation. The numbers adjacent to the outlined areas indicate the frequencies of IFN-γ^+^ cells in CD44^+^CD4^+^ T cells. **h, i**, Frequency (h) and number (i) of SARS-CoV-2 S1-stimulated IFN-γ^+^CD4^+^ T cells in g. PBS (n = 3 mice), NDV-WT (n = 3 mice) and NDV-RBD (n = 4 mice) in e, f, h, i. All the data are representative of two independent experiments. One-way ANOVA with Turkey’s test was used in e, f, h, i. Error bars (e, f, h, i) indicate s.e.m. Source data

Considering that a tremendous population around the globe has been either infected with SARS-CoV-2 or immunized with COVID-19 vaccines, both of which leave behind long-term persistence of SARS-CoV-2-specific T cell memory^44^, we set out to determine whether such T cell memory could be used by OV-BYTE therapy. To this end, naive BALB/c mice were prime–boost intranasally vaccinated with SARS-CoV-2 receptor-binding domain (RBD) proteins, a core part of the SARS-CoV-2 spike protein and COVID-19 vaccines^45^, and thus gained CD8^+^ and CD4^+^ T cell memory specific to the SARS-CoV-2 RBD (Extended Data Fig. 9a,b). Next, these vaccinated mice received orthotopic injection of murine triple-negative breast cancer 4T1 cells into mammary fat pads on day 60 after vaccination and then were intratumorally treated with an NDV stain expressing SARS-CoV-2 RBD (NDV-RBD). Consistently, intratumoral administration of NDV-RBD protected against the outgrowth of 4T1 tumors in SARS-CoV-2 RBD protein-immunized mice (Fig. 8a–c), with increased SARS-CoV-2 RBD-specific CD4^+^ T_BYS_ cells in the TME (Extended Data Fig. 9c–i). Furthermore, the protection against 4T1 tumors was reinforced when PD-L1 ICB was given concomitantly with NDV-RBD (Fig. 8a–c).

**Extended Data Fig. 10:**
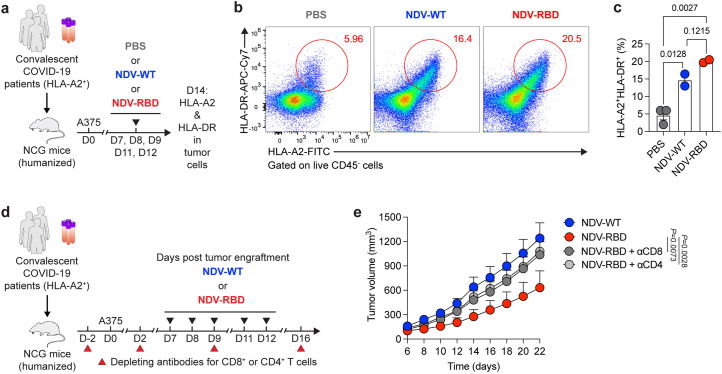
Both CD8^+^ and CD4^+^ T cells are indispensable for the anti-tumor function of OV-BYTE therapy in humanized NCG mice. **a**, Schematic experimental design. NCG mice were engrafted with PBMCs from the HLA-A2-positive convalescent COVID-19 patients. Reconstructed humanized NCG mice were then engrafted with A375 cells and then intratumorally administrated with PBS or NDV-WT or NDV-RBD on day 7, 8, 9, 11 and 12 after tumor engraftment. On day 14-post tumor engraftment, HLA-A2 and HLA-DR expression levels were detected in A375 tumor cells. **b**, Flow cytometry analysis of HLA-A2^+^HLA-DR^+^ cells in CD45^−^ cells from PBS- or NDV-WT- or NDV-RBD-treated A375 tumors as described in a. The numbers adjacent to the outlined areas indicate the frequencies of HLA-A2^+^HLA-DR^+^ cells in CD45^−^ cells. **c**, Frequencies of HLA-A2^+^HLA-DR^+^ cells in CD45^−^ cells from PBS- (n = 3 mice) or NDV-WT- (n = 2 mice) or NDV-RBD-treated (n = 2 mice) A375 tumors. **d**, Schematic experimental design. NCG mice were engrafted with PBMCs from the HLA-A2-positive convalescent COVID-19 patients. Then, reconstructed humanized NCG mice were engrafted with A375 cells and then intratumorally administrated with NDV-WT or NDV-RBD on day 7, 8, 9, 11 and 12 after tumor engraftment. Meanwhile, recipients were intraperitoneally injected with depleting antibodies for CD8^+^ or CD4^+^ T cells on indicated time points. **e**, Tumor growth curve of A375 tumor-bearing NCG mice intratumorally treated with NDV-WT or NDV-RBD in the presence or absence of depleting antibody for CD8^+^ or CD4^+^ T cells. NDV-WT (n = 5 mice), NDV-RBD (n = 6 mice), NDV-RBD/αCD8 (n = 6 mice) and NDV-RBD/αCD4 (n = 5 mice). All the data are representative of two independent experiments. One-way ANOVA with Turkey’s test was used in c. Two-way ANOVA was used to compare tumor growth curves in e. Error bars (c, e) indicate s.e.m. Source data

To further probe the clinical relevance of OV-BYTE therapy leveraging SARS-CoV-2-specific T cell memory, NCG mice were engrafted with PBMCs from convalescent HLA-A2-typed patients with COVID-19 harboring SARS-CoV-2 RBD-specific T_MEM_ cells^46^ (Fig. 8d). These NCG mice were inoculated with A375 human melanoma cells and then intratumorally administered NDV-WT or NDV-RBD (Fig. 8e). Noticeably, A375 tumor growth was more restricted in the NDV-RBD-treated group than that in the NDV-WT-treated one (Fig. 8f). Recapitulating NDV treatment-upregulated MHC-I and MHC-II expression in murine tumor cells (Extended Data Fig. 4e,f), both MHC-I (HLA-A2) and MHC-II (HLA-DR) expression levels of A375 tumor cells were reinforced upon NDV treatment (Extended Data Fig. 10a–c). Furthermore, the therapeutic effects of NDV-RBD in A375 tumors engrafted in NCG mice receiving transfer of convalescent COVID-19 patient PBMCs were almost diminished upon CD8^+^ or CD4^+^ T cell depletion (Extended Data Fig. 10d,e). These findings indicate that both CD8^+^ and CD4^+^ T cells contribute to the anti-tumor functions of OV-BYTE therapy using SARS-CoV-2-specific T cell memory in the humanized NCG model.

We next used replication-deficient Ad5 virus expressing SARS-CoV-2 RBD (Ad5-RBD) to treat A375 tumors inoculated in NCG mice reconstructed by convalescent COVID-19 patient PBMCs (Fig. 8g). Consistently, Ad5-RBD delayed A375 tumor growth as compared to Ad5-WT (Fig. 8h). Therefore, these results together indicate that SARS-CoV-2-specific T cell memory established by infection or vaccination could be exploited by the OV-BYTE strategy to treat cancer.

## Discussion

In this study, we provided a proof of concept that redirecting the antigen specificity of tumor cells to tumor-infiltrating CD8^+^ and CD4^+^ T_BYS_ cells effectively controls tumor progression. This therapy, called OV-BYTE, was proven to curtail tumor growth and show synergistic effects with PD-L1 ICB therapy in multiple preclinical tumor models. In particular, we identified that TCF-1^lo^T-bet^hi^ cytotoxic CD4^+^ T_BYS_ cells conveyed GzmB-dependent cytotoxicity and were amenable to OV-BYTE-mediated therapeutic effects, thus being aligned with the recently highlighted cytotoxic functions of CD4^+^ T cells in eliminating cancer cells^47^ and virus-infected cells^48^.

We also provided key proof-of-concept data illustrating that OV-BYTE therapy can effectively harness SARS-CoV-2-specific T cell memory to treat cancers in preclinical models. Notably, several SARS-CoV-2-infected patients with cancer have been reported to achieve partial or complete tumor remission^49,50^. Based on our findings, we speculate that SARS-CoV-2 infection of tumor cells^51,52^ in these patients may redirect these malignant cells toward the cytotoxicity of SARS-CoV-2-specific T_BYS_ cells. Further clinical trials are urgently needed to validate the therapeutic efficacies of OV-BYTE therapy in patients with cancer and SARS-CoV-2-specific T cell memory.

In conclusion, we revealed that OV-BYTE therapy redirects the cytotoxicity of functional T_BYS_ cells toward tumor cells for improved tumor control and has synergistic effects with PD-L1 ICB. Hence, the combination of OV-BYTE and PD-1 and/or PD-L1 ICB might expand the toolkits for cancer immunotherapy.

## Methods

Research involving animal experiments with all relevant ethical regulations is detailed in Mice below. Research involving human samples with all relevant ethical regulations is detailed in Human participants and the humanized mouse model below.

### Mice

C57BL/6, BALB/c, OT-I and CD45.1^+^ (B6.SJL-*Ptprc*^*a*^ *Pepc*^*b*^/BoyJ) mice were purchased from the Jackson Laboratories. P14 and SM mice were gifts from R. Ahmed (Emory University). CXCR5–GFP knockin mice were generated by inserting an IRES-GFP construct after the open reading frame of *Cxcr5* (ref. ^53^). *Gzmb*^KO^ mice were provided by F. Shao (Chinese Academy of Medical Sciences and National Institute of Biological Sciences). All experiments involving C57BL/6, BALB/c, P14, SM, OT-I, CXCR5–GFP knockin, CD45.1^+^ and *Gzmb*^KO^ mice were carried out in accordance with procedures approved by the Institutional Animal Care and Use Committees of the Third Military Medical University. Immunodeficient NOD/ShiLtJGpt-*Prkdc*^*em26Cd52*^*Il2rg*^*em26Cd22*^/Gpt (NCG) mice were purchased from GemPharmatech. All experiments involving the generation and characterization of humanized NCG mice were approved by an institutional animal care and use committee at the Model Animal Research Center at Nanjing University (AP LY-01). All mice used for animal experiments were 6–10 weeks of age, and both sexes were included in the study, except only female BALB/c mice were used for the engraftment of 4T1 breast cancer cells. All mice were housed under specific-pathogen free conditions with normal mouse chow (Jiangsu Xietong Shengwu), a 12-h light–12-h dark cycle and controlled temperature (20–22 °C) and humidity (40–60%).

### Viral infection and protein immunization

To establish LCMV Armstrong infection, naive C57BL/6 mice were injected intraperitoneally with 2 × 10^5^ plaque-forming units (PFU) of LCMV Armstrong. To establish SARS-CoV-2-specific T cell memory, naive BALB/c mice were intranasally immunized with two doses of 10 μg SARS-CoV-2 RBD protein–mFC tag (Sino Biological, 40592-V05H) conjugated to 10 μg CpG ODN 1826 (Invitrogen, tlrl-1826) at an interval of 14 d.

### Construction of modified oncolytic viruses

NDV-LCMV GP, NDV-LCMV NP, NDV-SARS-CoV-2 RBD and NDV-H1N1 NP were generated as previously described^54^; specifically, the full-length NDV SG10 cDNA clone, pOK-SG10, was used as the backbone to construct a recombinant cDNA clone containing the gene sequences encoding LCMV GP, LCMV NP, SARS-CoV-2 RBD or H1N1 NP between the P and M genes. Ad5-GP and Ad5-RBD were generated by inserting the gene sequences encoding LCMV GP protein and SARS-CoV-2 RBD protein, respectively, into Ad5 lacking 24 base pairs encoding eight amino acids in the pRb-binding domain of E1A and E3.

### Tumor models

MC38 (NM-S13) and MC38-OVA (NM-S13-TM56) cells were purchased from the Shanghai Model Organisms Center. B16F10 (CRL-6475), 4T1 (CRL-2539) and A375 (CRL-1619) cells were purchased from ATCC. B16-GP cells were generated by CRISPR–Cas9-mediated insertion of the LCMV GP gene into the genome of B16F10 cells. All tumor cell lines were cultured in complete DMEM-10 medium (Gibco) supplied with 10% FBS (Gibco), 1% penicillin–streptomycin (Gibco) and 1% l-glutamine (Gibco). For subcutaneous tumor models, C57BL/6 mice were implanted with 3–5 × 10^5^ MC38, MC38-OVA, B16F10 or B16-GP cells in the left flank of leg. For the mouse breast cancer model, 2 × 10^5^ 4T1 cells were implanted into the mammary fat pad of female BALB/c mice. Tumor volumes were determined in two dimensions with a caliper and calculated according to the formula ((length × width^2^)/2). For the NICD and AKT plasmid-induced intrahepatic cholangiocarcinoma model, the hydrodynamic tail vein of C57BL/6 mice was injected with a mixture of the three plasmids, including Myc-tagged NICD1 (10 μg per mouse), Myc-tagged AKT (4 μg per mouse) and SB100 (1 μg per mouse), according to a published study^35^. For the tumor transplantation model, MC38 tumors were surgically dissected from donor mice and cut into pieces of about 5 mm^3^ in size. Next, tumor specimens were subcutaneously transplanted into the same flank of recipients. Tumor-engrafted mice were killed at the indicated time points on the premise of humane endpoints (tumor volume not exceeding 2,000 mm^3^).

### PCR with reverse transcription

Total RNA from tumor cells was extracted by using TRIzol LS (Life Technologies) and reverse transcribed using the RevertAid Minus First Strand cDNA Synthesis Kit (Thermo Scientific, K1622) according to the manufacturer’s protocol. Relative expression levels of transcripts were determined using AceQ qPCR SYBR Green Master Mix (Vazyme, Q111) with a CFX96 Touch Real-Time System (Bio-Rad). The primers used in the study are as follows: LCMV GP gene (forward, 5′-GCAACTGCTGTGTTCCCGAAAC-3′; reverse, 5′-CATTCACCTGGACTTTGTCAGACTC-3′), LCMV NP gene (forward, 5′-CTCACACGGCATGGATCTTG-3′; reverse, 5′-TCGAAGCTTCCCTGGTCATT-3′) and *Hprt1* (forward, 5′-TCAGTCAACGGGGGACATAAA-3′; reverse, 5′-GGGGCTGTACTGCTTAACCAG-3′).

### Immunofluorescence

MC38 cells were transferred to 12-mm coverslips in a 12-well plate. After fixation and permeabilization, cells on coverslips were stained with DAPI (Sigma, D9542) and for actin (Cell Signaling Technology, 3134) and the NDV P protein (made in house). Images were acquired with a Zeiss LSM 510 confocal fluorescence microscope and processed with LSM Image Examiner (version 4.0).

### Adoptive transfer of T cells

For the LCMV Armstrong infection model, 5 × 10^4^ congenic naive splenic P14 cells and/or SM cells were adoptively transferred into recipients 1 d before infection. For the subcutaneous B16-GP tumor model, 5 × 10^5^ congenic naive splenic P14 cells and/or SM cells were adoptively transferred into recipients 1 d before B16-GP tumor engraftment. For the LCMV Armstrong-specific T_MEM_ cell transfer experiment, 4 × 10^6^ CD8^+^ and/or CD4^+^ T cells with LCMV memory were isolated from the spleens of infected mice at day 60 after LCMV Armstrong infection and then adoptively transferred into naive C57BL/6 recipients. For the MC38-OVA tumor model, 2 × 10^3^ congenic OT-I cells were adoptively transferred into recipients on day 10 after MC38-OVA tumor engraftment.

### Isolation of lymphocytes

Lymphocytes in spleens were collected by mashing spleens through a cell strainer (BD Falcon). Lymphocytes in the peripheral blood were obtained by using a Ficoll (TBD, LTS107701) density gradient. To obtain liver-resident lymphocytes, mice were euthanized and perfused. Next, livers were dissected and mechanically minced. Liver-resident lymphocytes were acquired using a Percoll (GE Healthcare, 17-0891-09) density gradient. To obtain MC38, MC38-OVA or 4T1 tumor-infiltrating lymphocytes (TILs), tumors were mechanically minced, digested using with 1 mg ml^−1^ collagenase I (Sigma, c0130) and mashed through filters. Next, we enriched for TILs using a Percoll (GE Healthcare, 17-0891-09) density gradient. To collect B16F10 or B16-GP TILs, tumors were mechanically minced, and we enriched for TILs using a Percoll (GE Healthcare, 17-0891-09) density gradient.

### Human participants and the humanized mouse model

We enrolled a cohort containing eight HLA-A2-positive healthy volunteers and a cohort containing four HLA-A2-positive convalescent patients with COVID-19. All volunteers provided written informed consent. The study received institutional review board approvals at Chongqing Public Health Medical Center (2020-023-01-KY). PBMCs from blood donated by these volunteers were isolated using a Ficoll (TBD, LTS107701) density gradient. NCG mice were injected intravenously with 5 × 10^6^ human PBMCs. After the humanization rate reached an appropriate proportion, 5 × 10^6^ A375 cells were subcutaneously injected into each side of recipient mice in a volume of 100 µl PBS. Tumors were measured every day using calipers, and the tumor volume was calculated using the following formula: (length × width^2^)/2.

### Enzyme-linked immunosorbent assay

ELISA plates (Thermo Fisher, 446469) were coated with 50 ng H1N1 NP protein (Sino Biological, 40777-V08B) in 100 μl PBS per well overnight. Next, the ELISA plates were incubated with blocking buffer (5% FBS with 0.1% Tween-20 in PBS) for 1 h. Fifty-fold-diluted sera or blocking buffer (negative control) were next added to each well and incubated for 1 h. The ELISA plates were then washed with PBST (PBS with 0.1% Tween-20), incubated with HRP-conjugated goat anti-human IgG antibody (Bioss Biotech) and washed with PBS, and TMB was added (Beyotime). Samples were allowed to react with TMB for ~5 min, and then the reaction in ELISA plates was stopped by adding 1 M H_2_SO_4_ stop buffer. Optical density values were determined at 450 nm.

### Administration of reagents

For administration of OVs, mice were treated intratumorally with 2 × 10^6^ PFU of NDV or 1 × 10^8^ PFU of Ad5 in 50 μl PBS. For the LCMV GP-neutralizing antibody experiment, sera containing LCMV GP-neutralizing antibodies were collected from C57BL/6 mice infected with LCMV Cl13 (ref. ^55^), pathogen inactivated and then incubated in vitro with NDV-GP. For PD-L1 ICB experiments, mice were intraperitoneally injected with 150 μg anti-PD-L1 antibody (BioXCell, clone 10F.9G2) or an isotype-matched control antibody (BioXCell, clone LTF-2) on days 10, 13 and 16 after tumor engraftment. For the depletion of CD8^+^ or CD4^+^ T cells in C57BL/6 mice, mice were intraperitoneally injected with 100 μg anti-CD8 antibody (BioXCell, clone 53-6.7) or 100 μg anti-CD4 antibody (BioXCell, clone GK1.5) on days −1, 1, 7 and 12 after tumor engraftment. For the depletion of CD8^+^ or CD4^+^ T cells in humanized NCG mice, mice were intraperitoneally injected with 200 μg anti-CD8 antibody (BioXCell, clone OKT8) or 200 μg anti-CD4 antibody (BioXCell, clone OKT4) on days −2 and 2 and then further injected intraperitoneally with 50 μg anti-CD8 antibody or anti-CD4 antibody on days 9 and 16 after tumor engraftment. For FTY720 treatment, mice were intraperitoneally injected with 0.3 mg per kg FTY720 (Merck, SML0700).

### Flow cytometry

All information on antibodies for flow cytometry analysis in this study is provided in the Reporting Summary. Cell surface marker staining was performed in PBS containing 2% FBS. For the detection of cytokines, lymphocytes were stimulated for 6 h in the presence of the indicated peptide (0.2 μg ml^−1^ LCMV GP_33–41_, LCMV GP_66–77_, LCMV GP_91–101_, LCMV GP_118–125_, LCMV GP_276–286_, OVA_257–264_, OVA_323–339_ or p15E peptide) and anti-CD107a and anti-CD107b antibodies in an incubator at 37 °C. Alternatively, lymphocytes were incubated for 6 h with 1 μg ml^−1^ of an H1N1 NP peptide pool (Miltenyi Biotec, 130-097-278), for 18 h with 1 μg ml^−1^ of a SARS-CoV-2 S1 peptide pool (Sino Biological, PP003-A) or for 44 h with 1 μM recombinant SARS-CoV-2 RBD protein in an incubator at 37 °C. Brefeldin A and monensin (BD Biosciences) were added into the culture 4 h before detection. Intracellular cytokine staining for IL-2, IFN-γ and TNF-α was performed with the Cytofix/Cytoperm Fixation/Permeabilization Kit (BD Biosciences, 554714). Staining for T-bet, TCF-1, TOX, Eomes, Ki-67 and GzmB was performed with the Foxp3/Transcription Factor Staining Buffer Set (eBioscience, 00-5523). Dead cells were stained using the LIVE/DEAD Fixable Near-IR Dead Cell Stain Kit (Life Technologies). Flow cytometry data were acquired using FACSDiva software (version 7.0) in the FACSCanto II (BD Biosciences) or the FACSFortessa (BD Biosciences) and analyzed using FlowJo (version 10.4.0). Cell sorting experiments were performed with the BD FACSAria III (BD Biosciences).

### Ex vivo killing assay

In the experiment illustrating NDV-GP-mediated GP protein expression in MC38 cells, NDV-WT- and NDV-GP-infected MC38 cells were labeled with CellTrace Violet (Thermo Fisher) at 0.1 μM and 1 μM, respectively, and then mixed at a 1:1 ratio. Next, labeled MC38 cells (target) were mixed with LCMV Armstrong-activated or naive CD8^+^ T cells (effector) at a 100:1, 50:1 or 25:1 E:T ratio and co-cultured at 37 °C for 8 h. In experiments assessing the killing efficacy of SM T_BYS_ cells, NDV-WT-treated MC38 cells were labeled with CellTrace Violet (Thermo Fisher) at either 0.1 μM or 1 μM. MC38 cells labeled with 1 μM CellTrace Violet were pulsed with 2 μg LCMV GP_66–77_ peptide and then mixed with MC38 cells labeled with 0.1 μM CellTrace Violet at a 1:1 ratio. These labeled MC38 target cells were further mixed with sorted SM T_BYS_ cells or naive CD4^+^ T cells (effector) at a 9:1 E:T ratio and co-cultured at 37 °C for 8 h. The killing efficiency was determined as follows: 100 − (((percent MC38-GP with LCMV-specific T cells)/(percent control MC38 with LCMV-specific T cells))/((percent MC38-GP with naive T cells)/(percent control MC38 with naive T cells))) × 100.

### scRNA-seq library preparation, sequencing and data processing

scRNA-seq libraries were prepared using Chromium Next GEM Single Cell 3′ Reagent Kits (10x Genomics) according to the manufacturer’s instructions and then sequenced with paired-end sequencing of 150 nucleotides on each end on one lane of a NovaSeq 6000 per sample. Raw gene expression matrices were generated with the Cell Ranger (version 6.1.1) pipeline coupled with the mm10 genome annotation reference, which was then processed using Seurat^56^, and only cells with >1,000 detected genes or a mitochondrial read percentage <5 were retained. Doublets were automatically detected and removed by scDblFinder^57^ before all samples were combined into a count matrix using the merge function in Seurat. Data were log normalized and scaled using ‘NormalizeData’ and ‘ScaleData’. The top 2,000 most variable genes were calculated using ‘FindVariableGenes’ and then used in the PCA calculation with RunPCA. The number of top PCs used to calculate a UMAP dimensional reduction was decided by observation of the graphic output of the ‘ElbowPlot’ function.

### Identification of differentially expressed genes and functional enrichment

DEG testing was performed using the ‘FindMarkers’ function in Seurat with default parameters. The top 200 DEGs with the highest log_2_ (fold change) values and a maximum FDR value of 0.01 were filtered and exploited for the enrichment analysis, which was conducted using the Metascape webtool (https://www.metascape.org). Gene sets were derived from the GO Biological Process ontology.

### Defining cell state scores

We generated gene sets representing the signatures of T_CM_ cells and T_RM_ cells by conducting DEG identification analysis on external bulk RNA-seq datasets^31,32^. The resulting gene sets were used as input for the ‘AddModuleScore’ function in Seurat to calculate the corresponding cell scores, which could be further interpreted as phenotypic similarity to T_CM_ and T_RM_ for each cell.

### Consensus non-negative matrix factorization analysis

Data from P14 T_BYS_ cells (PBS group) were merged with data from P14 T_RM_ cells (kidney, liver, salivary gland, siIEL)^24^ and P14 T_MEM_ cells (days 32, 60 and 90)^25^ from an external dataset to generate a mixed count matrix first, which was the foundation for the inference of consensus gene programs. In detail, we executed the ‘cnmf prepare’ command from the cNMF package^58^ to normalize the input matrix and prepare the run parameters, the ‘cnmf factorize’ command to factorize the matrix and the ‘cnmf combine’ command to combine the individual spectrum result files for each *K* (the expected number of gene modules, which should be previously assigned by prior knowledge in the first step) into a merged file. The optimal number for *K* was decided by observing the output of the ‘cnmf k_selection_plot’ command, considering the tradeoff between stability and error, which distinguished the number 9 from the other candidates for the optimal value of *K*. At the desired value of *K*, we obtained consensus estimates for the programs and their usages by executing the ‘cnmf consensus’ command. Calculation and visualization using Radviz was based solely on the results of the cNMF analysis.

### RNA velocity analysis

Read annotations for sequenced samples were performed using the ‘velocity run 10X’ command-line tool with BAM (velocyto.py), genome annotation and repeat annotation^59^. Repeat annotation files were downloaded from the UCSC Genome Browser. The UMAP embedding matrix computed with the Seurat pipeline was used to construct the velocity map with the scVelo Python package^60^. Briefly, the total procedure involved execution of the pp.filtered_and_normalized, pp.moments, pp.neighbors, tl.recover_dynamics and tl.velocity (with the mode parameter set to ‘dynamical’) functions from the scVelo package.

### Bulk TCR amplification, sequencing and data analysis

RNA samples were analyzed by high-throughput sequencing of the TCR β-chain (TRB) using the ImmuHub TCR profiling system^61^ (ImmuQuad Biotech). The raw sequencing data were then aligned with NCBI and PCR. The resulting nucleotide and amino acid sequences of complementarity-determining region 3 (CDR3) of TRB were determined, and those with out-of-frame and stop codon sequences were removed from the identified TRB repertoire. We further defined the amount of each TRB clonotype by adding numbers of TRB clones sharing the same nucleotide sequence as CDR3. Shannon’s index^62^ was used to assess the diversity of TCR clones within each sample, which was defined as follows:

$$H^{\prime}=-\sum p_i{\mathrm{ln}}({p_i}),$$

where *p*_*i*_ defines the frequency of the *i*-th specific clonotype.

### Single-cell TCR sequencing data analysis

scTCR-seq libraries were prepared using 10x Genomics 5′ Single Cell Immune Profiling technology according to the manufacturer’s instructions. Sequencing was performed by using the NovaSeq 6000. TCR clonotype identification, alignment and annotation were performed using the 10x Genomics Cell Ranger pipeline (version 6.1.1) according to the manufacturer’s instructions. Clonotype alignment was performed against the Cell Ranger human V(D)J reference library 3.1.0 (GRCh38 and Ensembl GTF version 94). TCR clonotypes were defined using the ‘combineTCR’ function in scRepertoire^63^ (with the ‘cloneCall’ parameter set to ‘CTstrict’). Clonal expansion was determined using a five-group classification. The percentage of cells in single (0–1), small (2–5 cells), medium (6–10 cells), large (11–30) and hyperexpanded (31–70 cells) clones was determined for *Pdcd1*^+^*Entpd1*^+^*Cd8a*^+^ T cells.

### Statistics and reproducibility

No statistical method was used to predetermine sample size, but our sample sizes are similar to those reported in previous publications^19,27,28,53,64^. No data were excluded from the analyses. The experiments were not randomized. The investigators were not blinded to allocation during experiments and outcome assessment. Statistical analysis was performed with Prism 9.0 (GraphPad). Normality and equal variances of data were formally tested. For comparisons between two independent groups, two-tailed unpaired Student’s *t*-test was used. For comparisons among three or more groups, one-way ANOVA with Turkey’s test was used. Two-way ANOVA was used to compare tumor growth curves. The log-rank (Mantel–Cox) test was performed to compare survival curves among groups. Graphs show individual samples, and center values indicate the mean. *P* values less than 0.05 were defined as statistically significant.

### Reporting summary

Further information on research design is available in the Nature Portfolio Reporting Summary linked to this article.

### Supplementary information

Reporting Summary

### Source data

Source Data Fig. 1 Statistical source data for Fig. 1.

Source Data Fig. 2 Statistical source data for Fig. 2.

Source Data Fig. 3 Statistical source data for Fig. 3.

Source Data Fig. 4 Statistical source data for Fig. 4.

Source Data Fig. 5 Statistical source data for Fig. 5.

Source Data Fig. 6 Statistical source data for Fig. 6.

Source Data Fig. 7 Statistical source data for Fig. 7.

Source Data Fig. 8 Statistical source data for Fig. 8.

Source Data Extended Data Fig. 1 Statistical source data for Extended Data Fig. 1.

Source Data Extended Data Fig. 2 Statistical source data for Extended Data Fig. 2.

Source Data Extended Data Fig. 3 Statistical source data for Extended Data Fig. 3.

Source Data Extended Data Fig. 4 Statistical source data for Extended Data Fig. 4.

Source Data Extended Data Fig. 5 Statistical source data for Extended Data Fig. 5.

Source Data Extended Data Fig. 6 Statistical source data for Extended Data Fig. 6.

Source Data Extended Data Fig. 7 Statistical source data for Extended Data Fig. 7.

Source Data Extended Data Fig. 8 Statistical source data for Extended Data Fig. 8.

Source Data Extended Data Fig. 9 Statistical source data for Extended Data Fig. 9.

Source Data Extended Data Fig. 10 Statistical source data for Extended Data Fig. 10.

## Data Availability

scRNA-seq data of T_BYS_ cells and conventional memory SM cells, scRNA-seq and scTCR-seq data of PD-1^+^CD39^+^CD8^+^ T cells and TCR-seq data of p15E tetramer^+^CD8^+^ T cells in this study have been deposited in the Gene Expression Omnibus under accession code GSE222002. Public scRNA-seq and bulk RNA-seq data reanalyzed here are available under accession codes GSE182276, GSE131847 and GSE128197. All other data supporting the findings of this study are available from the corresponding authors upon reasonable request. Source data are provided with this paper.

## Code availability

There were no custom algorithms in this study. Open-source software was used to analyze data. Details of software versions are specified in the Methods.
