# Supplementary material for: An oncolytic virus delivering tumor-irrelevant bystander T cell epitopes induces anti-tumor immunity and potentiates cancer immunotherapy
Source: Nat Cancer. 2024 Apr 12;5(7):1063–81. doi: 10.1038/s43018-024-00760-x (PMC11286533; doi:10.1038/s43018-024-00760-x)
Supplement: Supplementary file 1 — Reporting Summary [file 43018_2024_760_MOESM1_ESM.pdf]

nature portfolio

Corresponding author(s): Lilin Ye

Last updated by author(s): Jan 29, 2024

Reporting Summary

Nature Portfolio wishes to improve the reproducibility of the work that we publish. This form provides structure for consistency and transparency in reporting. For further information on Nature Portfolio policies, see our Editorial Policies and the Editorial Policy Checklist.

Statistics

For all statistical analyses, confirm that the following items are present in the figure legend, table legend, main text, or Methods section.

n/a	Confirmed	
☐	☒	The exact sample size (*n*) for each experimental group/condition, given as a discrete number and unit of measurement
☐	☒	A statement on whether measurements were taken from distinct samples or whether the same sample was measured repeatedly
☐	☒	The statistical test(s) used AND whether they are one- or two-sided<br>*Only common tests should be described solely by name; describe more complex techniques in the Methods section.*
☒	☐	A description of all covariates tested
☐	☒	A description of any assumptions or corrections, such as tests of normality and adjustment for multiple comparisons
☐	☒	A full description of the statistical parameters including central tendency (e.g. means) or other basic estimates (e.g. regression coefficient) AND variation (e.g. standard deviation) or associated estimates of uncertainty (e.g. confidence intervals)
☐	☒	For null hypothesis testing, the test statistic (e.g. *F*, *t*, *r*) with confidence intervals, effect sizes, degrees of freedom and *P* value noted<br>*Give P values as exact values whenever suitable.*
☒	☐	For Bayesian analysis, information on the choice of priors and Markov chain Monte Carlo settings
☒	☐	For hierarchical and complex designs, identification of the appropriate level for tests and full reporting of outcomes
☒	☐	Estimates of effect sizes (e.g. Cohen's *d*, Pearson's *r*), indicating how they were calculated

Our web collection on statistics for biologists contains articles on many of the points above.

Software and code

Policy information about availability of computer code

Data collection	Flow cytometry data were collected by FACSDiva (version 7.0) software in FACSCanto II (BD Biosciences) or FACSFortesa (BD Biosciences). Single-cell sequencing data were collected using Cell Ranger software (10x Genomics) version 6.1.1. Immunofluorescence images were acquired with a Zeiss LSM 510 confocal fluorescence microscope.
Data analysis	Flow cytometry data were analyzed with FlowJo (version 10.4.0).<br><br>Statistical analysis was performed with Prism 9.0 (GraphPad).<br><br>For bioinformatic analyses, we primarily used existing computational tools, and we cited all the sources of the tools we used:<br>https://github.com/satijalab/seurat (v. 4.1.1);<br>https://github.com/plger/scDblFinder (v. 1.8.0);<br>https://github.com/theislab/scvelo (v. 0.2.4);<br>https://github.com/velocyto-team/velocyto.py (v. 0.17.17);<br>https://github.com/dylkot/cNMF (v. 1.4);<br>https://github.com/ncborcherding/scRepertoire (v. 1.8.0);<br>https://github.com/yannabraham/Radviz (v. 0.9.3).<br><br>Immunofluorescence images were processed with LSM Image Examiner (version 4.0).

For manuscripts utilizing custom algorithms or software that are central to the research but not yet described in published literature, software must be made available to editors and reviewers. We strongly encourage code deposition in a community repository (e.g. GitHub). See the Nature Portfolio guidelines for submitting code & software for further information.

Data

Policy information about availability of data

All manuscripts must include a data availability statement. This statement should provide the following information, where applicable:

- Accession codes, unique identifiers, or web links for publicly available datasets
- A description of any restrictions on data availability
- For clinical datasets or third party data, please ensure that the statement adheres to our policy

The scRNA-seq data of MC38 tumor-infiltrating P14/SM TBYS cells and conventional memory SM cells, the scTCR-seq data of MC38 tumor-infiltrating CD8+ T cells and the bulk TCR-seq data of MC38 tumor-infiltrating p15E-specific CD8+ T cells in this study have been deposited with GEO under accession code GSE222002 (https://www.ncbi.nlm.nih.gov/geo/query/acc.cgi?acc=GSE222002). Public scRNA-seq and bulk RNA-seq data reanalyzed here are available under accession codes (GSE182276, GSE131847 and GSE128197). Source data for Figures 1-8 and Extended Data Figures 1-10 have been provided as Source Data files.

Research involving human participants, their data, or biological material

Policy information about studies with human participants or human data. See also policy information about sex, gender (identity/presentation), and sexual orientation and race, ethnicity and racism.

Reporting on sex and gender	We enrolled a cohort containing 8 HLA-A2-positive healthy volunteers (4 female and 4 male) and a cohort containing 4 HLA-A2-positive convalescent COVID-19 patients (2 female and 2 male). Inclusion criteria was not sex-based but rather based on their established T cell memory to H1N1 and SARS-CoV-2 infections.
Reporting on race, ethnicity, or other socially relevant groupings	All the participants in this study were Chinese of HLA-A2 restriction and harbor T cell memory specific to H1N1 NP or SARS-CoV-2 RBD.
Population characteristics	Cohort 1: healthy volunteers, 4 female and 4 male, age range: 27-35, HLA-A2+, infection history of H1N1 as diagnosed by functional ELISA assays and H1N1 NP peptide stimulation assay;<br>Cohort 2: convalescent COVID-19 patients, 2 female and 2 male, age range: 36-50, HLA-A2+, infection history of SARS-CoV-2 during Feb., 2020 to Apr., 2020.
Recruitment	Healthy volunteers with a history of H1N1 infection (as diagnosed by ELISA assays) and H1N1 NP-specific T cell memory were recruited in the study (as suggested by H1N1 NP peptide stimulation assay). Convalescent COVID-19 patients (as diagnosed by ELISA assays) with SARS-CoV-2 RBD-specific T cell memory (as suggested by SARS-CoV-2 RBD protein stimulation assay) were recruited in the study.<br><br>Healthy donors or convalescent COVID-19 patients were recruited under Institutional Review Board approvals at Chongqing Public Health Medical Center (2020-023-01-KY).
Ethics oversight	The study received Institutional Review Board approvals at Chongqing Public Health Medical Center (2020-023-01-KY). All volunteers were provided written informed consent.

Note that full information on the approval of the study protocol must also be provided in the manuscript.

Field-specific reporting

Please select the one below that is the best fit for your research. If you are not sure, read the appropriate sections before making your selection.

☒ Life sciences ☐ Behavioural & social sciences ☐ Ecological, evolutionary & environmental sciences

For a reference copy of the document with all sections, see nature.com/documents/nr-reporting-summary-flat.pdf

Life sciences study design

All studies must disclose on these points even when the disclosure is negative.

Sample size	No statistical methods were used to predetermine the sample size. In most cases, each experimental group contains 4-6 biological replicates (flow cytometry, RT-PCR) or 8-12 biological replicates (tumor survival assay, scRNA-seq, scTCR-seq, bulk TCR-seq). The sample sizes in the study were empirically determined to optimized numbers necessary for statistical significance according to previous published studies of our own and other research groups (He, R. et al., Nature, 2016, PMID: 27501245; Khan, O. et al., Nature, 2019, PMID: 31207603; Huang, Q. et al., Cell, 2022, PMID: 36208623).
Data exclusions	No data were excluded from the analyses.
Replication	Except the construction of scRNA-seq/scTCR-seq/TCR-seq libraries, all the in vivo and in vitro experiments were performed independently at least 2 times. For the scRNA-seq/scTCR-seq/TCR-seq libraries, each library sample was originated from the pooled target cells of 8-12 mice. And the key scRNA-seq/scTCR-seq/TCR-seq data were validated by independent flow cytometry assays. All results described in the study can be reproduced.

Randomization	For animal experiments, control and treatment groups were delineated in each figure and figure legend. Before the treatment, tumor-engrafted mice were evenly distributed into each group based on the mean tumor volume of the whole cohort so that the standard deviation was equal across all groups.
Blinding	Experiments in the study were not performed in a blinded manner due to no involvement of subjective measurements. Besides, objective readouts were gained as experimental outcomes in the study. Thus, the data in the study were not prone to subjective evaluation.

Reporting for specific materials, systems and methods

We require information from authors about some types of materials, experimental systems and methods used in many studies. Here, indicate whether each material, system or method listed is relevant to your study. If you are not sure if a list item applies to your research, read the appropriate section before selecting a response.

Materials & experimental systems

n/a	Involved in the study
☐	☒ Antibodies
☐	☒ Eukaryotic cell lines
☒	☐ Palaeontology and archaeology
☐	☒ Animals and other organisms
☒	☐ Clinical data
☒	☐ Dual use research of concern
☒	☐ Plants

Methods

n/a	Involved in the study
☒	☐ ChIP-seq
☐	☒ Flow cytometry
☒	☐ MRI-based neuroimaging

Antibodies

Antibodies used

Flow cytometry antibodies used in mouse studies (company, cat number, clone, dilution):
anti-mouse CD8 (BioLegend, 100743, clone 53-6.7, 1:200), anti-mouse CD4 (BioLegend, 100553, clone RM4-5, 1:200), anti-mouse CD45 (BioLegend, 103122, clone 30-F11, 1:200), anti-mouse CD45.1 (BioLegend, 110706, clone A20, 1:200), anti-mouse Vα2 (BioLegend, 127810, clone B20.1, 1:200), anti-mouse MHC-I (BioLegend, 114614, clone 28-8-6), anti-mouse MHC-II (BioLegend, clone M5/114.15.2), anti-mouse F4/80 (BioLegend, 123110, clone BM8, 1:200), anti-mouse CD11b (BioLegend, 101211, clone M1/70, 1:200), anti-mouse CD11c (BioLegend, 117308, clone N418, 1:200), anti-mouse Ly-6C (BD Biosciences, 562737, clone AL-21, 1:200), anti-mouse H-2Kb bound to SIINFEKL antibody (BioLegend, 141606, clone 25-D1.16, 1:200), anti-mouse KLRG1 (BD Biosciences, 561621, clone 2F1, 1:200), anti-mouse CD127 (BioLegend, 158204, clone A7R34, 1:200), anti-mouse CD44 (BioLegend, 103047, clone IM7, 1:200), anti-mouse CD62L (BioLegend, 104418, clone MEL-14, 1:200), anti-mouse PD-1 (BioLegend, 135219, clone 29F.1A12, 1:200), anti-mouse Tim-3 (R&D systems , FAB1529P, clone 215008, 1:200), anti-mouse LAG-3 (BioLegend, 125208, clone C9B7W, 1:200), anti-mouse 2B4 (BioLegend, 133504, clone m2B4 (B6)458.1, 1:200), anti-mouse CD39 (BioLegend, 143804, clone Duha59, 1:200), anti-mouse Ly108 (BioLegend, 134610, clone 330-AJ, 1:200), anti-mouse GITR (BioLegend, 126312, clone DTA-1, 1:200), anti-mouse CD69 (BioLegend, 104512, clone H1.2F3, 1:200), anti-mouse CD103 (BioLegend, 121418, clone 2E7, 1:200), anti-mouse CD25 (BioLegend, 102028, clone PC61, 1:200), H-2Kb MuLV p15E Tetramer-KSPWFTTL-PE (MBL, TS-M507-1, 1:10), anti-mouse CD107a (BioLegend, 121606, clone 1D4B, 1:400), anti-mouse CD107b (BioLegend, 108504, clone M3/84, 1:400), anti-mouse IL-2 (BioLegend, 503824, clone JES6-5H4, 1:50), anti-mouse IFN-γ (BD Biosciences, 563376, clone XMG1.2, 1:100), anti-mouse TNF-α (BioLegend, 506108, clone TN3-19.12, 1:100), anti-mouse TCF-1 (Cell Signaling Technology, 6444S, clone C63D9, 1:400), donkey anti-rabbit polyclonal IgG (H+L) highly cross-adsorbed secondary antibody (Thermo Fisher, A-21206, 1:1000), anti-mouse Eomes (eBioscience, 25-4875-82, clone Dan11mag, 1:100), anti-mouse T-bet (BioLegend, 644808, clone 4B10, 1:100), anti-mouse TOX (Thermo Fisher, 12-6502-82, TXRX10, 1:100), anti-mouse Ki67 (BD Biosciences, 556027, 1:20) and anti-mouse GZMB (BioLegend, 372208, clone QA16A02, 1:100).

Flow cytometry antibodies used in human studies (company, cat number, clone, dilution):
anti-human CD45 (BioLegend, 304039, clone HI30, 1:200), anti-human CD8 (BioLegend, 300922, clone HIT8a, 1:200), anti-human CD4 (BioLegend, 317444, clone OKT4, 1:200), anti-human TNF-α (BioLegend, 376206, clone W19063E, 1:200), anti-human HLA-A2 (BioLegend, 343304, clone BB7.2, 1:200) and anti-human HLA-DR (BioLegend, 307617, clone L243, 1:200).

Blocking or depleting antibodies:
anti-mouse PD-L1 (BioXcell, clone 10F.9G2), isotype-matched control antibody (BioXcell, rat IgG2b, clone LTF-2), anti-mouse CD8 antibody (BioXell, clone 53-6.7), anti-mouse CD4 antibody (BioXell, clone GK1.5), anti-human CD8 antibody (BioXCell, clone OKT8), anti-human CD4 antibody (BioXCell, clone OKT4).

Antibodies for immunofluorescence or ELISA assays:
anti-actin (Cell Signaling Technology, 3134), anti-NDV P protein (provide by Dr. Guozhong Zhang, China Agricultural University), HRP-conjugated goat anti-human IgG antibody (Bioss Biotech).

Validation

The validation data of all the commercial antibodies can be found on the manufactures' websites.

Antibody (rabbit polyclonal antibody) specific to NDV P protein was provide by Dr. Guozhong Zhang (China Agricultural University) and was validated by previously published study (Xiao Li et al., Virulence, 2023, PMID: 36919461).

Eukaryotic cell lines

Policy information about cell lines and Sex and Gender in Research

Cell line source(s)	MC38 cells (NM-S13) and MC38-OVA (NM-S13-TM56) were purchased from Shanghai Model Organisms Center. B16F10 cells (CRL-6475), 4T1 cells (CRL-2539) and A375 cells (CRL-1619) were purchased from ATCC. B16-GP cells were purchased from Beijing Biocytogen Co.Ltd, China.
Authentication	B16-GP cell line was authenticated by PCR assays with species-specific primers (Huang, Q. et al., Cell, 2022, PMID: 36208623; Xiao, M. et al, J Immunother Cancer, 2022, PMID: 35580929). The other commercial cell lines were authenticated by Shanghai Model Organisms Center or ATCC and validated by PCR assays with species-specific primers in our lab.
Mycoplasma contamination	All the cell lines in the study were tested negative for mycoplasma contamination.
Commonly misidentified lines (See ICLAC register)	There were no misidentified lines in the study.

Animals and other research organisms

Policy information about studies involving animals; ARRIVE guidelines recommended for reporting animal research, and Sex and Gender in Research

Laboratory animals	C57BL/6, BALB/c, OT-I transgenic and CD45.1+ congenic (strain B6.SJL-Ptprca Pepcb/BoyJ) mice were purchased from the Jackson Laboratories. P14 transgenic (carrying a transgenic T cell antigen receptor that recognizes H-2Db GP33-41 epitope) and SMARTA transgenic (carrying a transgenic T cell antigen receptor that recognizes I-Ab GP66-77 epitope) mice were gifts from Dr. Rafi Ahmed (Emory University). The CXCR5-GFP knock-in mice were were purchased from Beijing Biocytogen Co.Ltd, China. Immunodeficient NOD/ShiLtJGpt-Prkdcem26Cd52Il2rgem26Cd22/Gpt (NCG) mice were purchased from the GemPharmatech Co., Ltd of Nanjing. Mice of 6-10-week-old were used for animal experiments.
Wild animals	There were no wild animals used in the study.
Reporting on sex	Both sexes were randomly allocated to into control and treatment groups in the study, except only female BALB/c mice were used for the engraftment of 4T1 breast cancer cells.
Field-collected samples	There were no samples collected from the field in the study.
Ethics oversight	All experiments involving C57BL/6, BALB/c, P14, SMARTA, OT-I, CXCR5-GFP knock-in and CD45.1+ congenic mice were carried out in accordance with procedures approved by the Institutional Animal Care and Use Committees of Third Military Medical University. All experiments involving the generation and characterization of humanized NCG mice were approved by an Institutional Animal Care and Use Committee (IACUC) at the Model Animal Research Center in Nanjing University (AP# LY-01).

Note that full information on the approval of the study protocol must also be provided in the manuscript.

Plants

Seed stocks	Does not apply in the study.
Novel plant genotypes	Does not apply in the study.
Authentication	Does not apply in the study.

Flow Cytometry

Plots

Confirm that:

☒ The axis labels state the marker and fluorochrome used (e.g. CD4-FITC).

☒ The axis scales are clearly visible. Include numbers along axes only for bottom left plot of group (a 'group' is an analysis of identical markers).

☒ All plots are contour plots with outliers or pseudocolor plots.

☒ A numerical value for number of cells or percentage (with statistics) is provided.

Methodology

Sample preparation	Lymphocytes in spleens were harvested by mashing the spleens through cell strainer (BD Falcon). Lymphocytes in the peripheral blood were obtained by using FICOLL (TBD, LTS107701) density gradient. To obtain liver-resident lymphocytes, mice were euthanized and perfused. Then, livers were dissected and mechanically minced. Liver-resident lymphocytes were acquired using Percoll (GE Healthcare, 17-0891-09) density gradient. To obtain MC38 or MC38-OVA or 4T1 tumor-infiltrating lymphocytes (TILs), tumors were mechanically minced, digested using with 1 mg/mL collagenase I (Sigma, c0130) and mashed through filters. Then, TILs were enriched using Percoll (GE Healthcare, 17-0891-09) density gradient. To harvest B16F10 or B16F10-GP TILs, tumors were mechanically minced and TILs were enriched using Percoll (GE Healthcare, 17-0891-09) density gradient. To obtain suspended tumor cells of A375 melanoma, tumors were mechanically minced, digested using with 1 mg/mL collagenase I (Sigma, c0130) and mashed through filters.
Instrument	Flow cytometry data were acquired using FACSCanto II (BD Biosciences) or FACSFortesa (BD Biosciences). Cell sorting experiments were performed with BD FACSAria III (BD Biosciences).
Software	BD FACSDiva software was used for data acquisition and FlowJo (version 10.4.0) was used for data analysis.
Cell population abundance	For the determination of sample purity, sorted target cells were collected by the BD FACSAria III (BD Biosciences) using the same gating strategy. And the purity of the collecting gate reached at least 95%.
Gating strategy	The cells were gated on FSC-A/SSC-A basis on the location known to immune cells or tumor cells, followed by excluding dead cells using live-dead dye negative gating. Next, the doublets were excluded by FSC-W/FSC-H and SSC-W/SSC-H. Then, target immune cells (e.g., T cells, DCs, macrophages) and tumor cells were gated according to well-known lineage and functional markers for further analyses.

☒ Tick this box to confirm that a figure exemplifying the gating strategy is provided in the Supplementary Information.
